# Practice Effects of Mobile Tests of Cognition, Dexterity, and Mobility on Patients With Multiple Sclerosis: Data Analysis of a Smartphone-Based Observational Study

**DOI:** 10.2196/30394

**Published:** 2021-11-18

**Authors:** Tim Woelfle, Silvan Pless, Andrea Wiencierz, Ludwig Kappos, Yvonne Naegelin, Johannes Lorscheider

**Affiliations:** 1 Research Center for Clinical Neuroimmunology and Neuroscience Basel University of Basel, University Hospital Basel Basel Switzerland; 2 Department of Neurology University Hospital Basel Basel Switzerland; 3 Clinical Trial Unit Department of Clinical Research University of Basel, University Hospital Basel Basel Switzerland

**Keywords:** multiple sclerosis, digital biomarkers, practice effects, learning effects, learning curves, nonlinear mixed models, quantile regression, information processing speed, symbol digit modalities test, smartphones, wearable electronic devices, mobile phones

## Abstract

**Background:**

Smartphones and their built-in sensors allow for measuring functions in disease-related domains through mobile tests. This could improve disease characterization and monitoring, and could potentially support treatment decisions for multiple sclerosis (MS), a multifaceted chronic neurological disease with highly variable clinical manifestations. Practice effects can complicate the interpretation of both improvement over time by potentially exaggerating treatment effects and stability by masking deterioration.

**Objective:**

The aim of this study is to identify short-term learning and long-term practice effects in 6 active tests for cognition, dexterity, and mobility in user-scheduled, high-frequency smartphone-based testing.

**Methods:**

We analyzed data from 264 people with self-declared MS with a minimum of 5 weeks of follow-up and at least 5 repetitions per test in the Floodlight Open study, a self-enrollment study accessible by smartphone owners from 16 countries. The collected data are openly available to scientists. Using regression and bounded growth mixed models, we characterized practice effects for the following tests: *electronic Symbol Digit Modalities Test (e-SDMT)* for cognition; *Finger Pinching* and *Draw a Shape* for dexterity; and *Two Minute Walk*, *U*-*Turn*, and *Static Balance* for mobility.

**Results:**

Strong practice effects were found for *e-SDMT* (n=4824 trials), *Finger Pinching* (n=19,650), and *Draw a Shape* (n=19,019) with modeled boundary improvements of 40.8% (39.9%-41.6%), 86.2% (83.6%-88.7%), and 23.1% (20.9%-25.2%) over baseline, respectively. Half of the practice effect was reached after 11 repetitions for *e-SDMT*, 28 repetitions for *Finger Pinching*, and 17 repetitions for *Draw a Shape*; 90% was reached after 35, 94, and 56 repetitions, respectively. Although baseline performance levels were highly variable across participants, no significant differences between the short-term learning effects in low performers (5th and 25th percentile), median performers, and high performers (75th and 95th percentile) were found for *e-SDMT* up to the fifth trial (β=1.50-2.00). Only small differences were observed for *Finger Pinching* (β=1.25-2.5). For *U*-*Turn* (n=15,051) and *Static Balance* (n=16,797), only short-term learning effects could be observed, which ceased after a maximum of 5 trials. For *Two Minute Walk* (n=14,393), neither short-term learning nor long-term practice effects were observed.

**Conclusions:**

Smartphone-based tests are promising for monitoring the disease trajectories of MS and other chronic neurological diseases. Our findings suggest that strong long-term practice effects in cognitive and dexterity functions have to be accounted for to identify disease-related changes in these domains, especially in the context of personalized health and in studies without a comparator arm. In contrast, changes in mobility may be more easily interpreted because of the absence of long-term practice effects, even though short-term learning effects might have to be considered.

## Introduction

### Background

Multiple sclerosis (MS) is a multifaceted and variable chronic autoimmune neurological disease affecting approximately 2.3 million people worldwide [1]. It is among the most common causes of nontraumatic disabilities in young adults [2].

MS progresses in different phases with highly variable speed and severity. To optimize treatment strategies, timely and precise monitoring of patients’ disease status is essential. As MS affects multiple functional domains, a range of validated clinical tests are used: for cognition, the Symbol Digit Modalities Test (SDMT) measures mental processing speed and is highly established as a screening tool for cognitive impairment in MS [3,4]. The 9-hole peg test (9HPT) is routinely used to measure dexterity [5,6], and the timed 25-foot walk (T25FW) is used to measure mobility [7]. Usually, stable patients with MS have half-yearly to yearly clinical routine visits with neurologic examinations and regular magnetic resonance imaging (MRI), limiting insight into symptom fluctuations and reversible deficits [8].

Wearable technologies, such as smartphones and smartwatches, are expected to capture more representative data at a higher resolution not only in the patients’ natural environments in MS but also in other neurological diseases such as Parkinson disease and Huntington disease [9-11]. Data can be collected passively during the patient’s everyday activities (eg, capturing step counts, turn speed, or keyboard dexterity [9,12,13]) or actively during specific functional tests [14-16]. They can possibly improve both clinical trials by providing more sensitive outcome measures and clinical practice by allowing more personalized disease course monitoring [14,17].

Acknowledged difficulties in interpreting the results of repeated tests are learning and practice effects, especially in neuropsychology [18,19]. Without a comparator, it is difficult to disentangle whether longitudinal improvement constitutes remission, practice, or treatment effects. In the same light, disease progression and worsening of disability may be masked by practice effects when specific tests feign stability. These issues have been adequately addressed by control groups in randomized controlled trials [20]. For trials without control groups and for intraindividual comparisons—a cornerstone of personalized medicine—interpretation remains challenging. Furthermore, practice effects hamper test-retest reliability, which is illustrated by recommendations to discard the results from 3 prebaseline repetitions of the MS functional composite [21]. However, more recently, it has been suggested that person-specific learning curves can be used as new outcome measures, leveraging the information inherent in practice effects [14].

### Objective

The aim of this analysis is to examine short-term learning and long-term practice effects in high-frequency smartphone-based tests representative of the assessment of 3 domains often affected by MS: cognition, dexterity, and mobility.

## Methods

### Study Data and Participant Selection

We used publicly available data from the *Floodlight Open* study, which collects smartphone-based test data from self-declared persons with MS with a number of different tests implemented in the Floodlight Open app [22]. The study is the successor of a small, closed feasibility study [9,17], and the data are openly available to researchers [23]. Currently, several phase 3 studies are using variations of the Floodlight app as part of their test batteries, for example, the CONSONANCE trial, a single-arm interventional trial evaluating ocrelizumab treatment in participants with progressive MS (NCT03523858) [17]. Recruitment for *Floodlight Open* started in April 2018, and some participants have been using the app continuously since then, amounting to more than 3 years of follow-up. However, most patients have only used the app for a very short time, leading to a strong right-skewness of the distribution of follow-up times. Among the 1147 patients who have performed at least one smartphone-based *e*-S*DMT* test in the period we examined, the median number of repetitions was 2, the IQR was 1-4 and the range was 1-119.

We included data up to and including July 31, 2021, and focused our analyses on the following 6 tests [9]: *e-SDMT* for cognition; *Finger Pinching* and *Draw a Shape* for dexterity; *Two Minute Walk*, *U-Turn*, and *Static Balance* for mobility. The dexterity tests have been shown to correlate with the 9HPT, the first 2 mobility tests with the T25FW and *Static Balance* with the Berg Balance Scale [9]. The Floodlight Open app allows performing *e-SDMT* up to a weekly frequency and all other tests up to a daily frequency, but the actual frequency was completely determined by the participant’s choice. For dexterity tests, the left and right hands were alternated.

The *e-SDMT* consisted of consecutively tapping symbol-corresponding digits on a number pad on the smartphone screen as quickly as possible for 60 seconds. Thus, there was a dexterity component that may potentially introduce bias. Floodlight’s *e-SDMT* included a second step termed *baseline*, simply showing digits instead of symbols, asking users to consecutively tap these digits on the same number pad for 15 seconds, without the symbol-association task. Using this second step by taking the quotient of the correct responses of the main test and the *baseline* potentially corrects for dexterity and reaction speed, representing only the true information processing speed.

Participants were selected for each test separately if at least 5 repetitions per test and at least 5 weeks between their first and last repetitions were available. This yielded slightly different but largely overlapping subsets of participants for each test.

### Statistical Analysis

#### Short-term Learning and Long-term Practice Effects

First, summary analyses were performed to investigate the mean scores of the first, fifth, and last trials of each test. We assumed that improvements up to the fifth score were more likely due to short-term learning effects, where participants learned to execute a test, and improvements from the fifth trial onward were more likely because of long-term practice effects. Naturally, these effects are intertwined, but using the fifth trial as the baseline was supported by Solari et al [24].

To examine group differences in baseline performances and potential short-term learning effects in low and high performers, linear quantile regression was performed on each test for the first 5 trials for the 5th, 25th, 50th, 75th, and 95th percentiles. Quantile regression *P* values were corrected with the Bonferroni method, and the 5 slopes were compared with an analysis of variance (ANOVA)–type test.

Long-term practice effects were assumed for tests with a significant mean difference from the fifth to the last score. The positive association of this difference with the number of repetitions (log-transformed to account for the strong right-skewness) adjusted for the potential confounders, age, first score, and fifth score, was considered as an additional indicator of long-term practice effects.

#### Long-term Learning Curve Analysis

For tests suggestive of long-term practice effects that meet the 2 abovementioned criteria, learning curve analysis was performed with 1 nonparametric and 3 parametric mixed effect models of increasing complexity, each modeling performance as a function of repetition, grouping by patient for cognition and mobility and by hand for dexterity. The performance of the 4 models was compared using both root mean squared error (RMSE) and the number of (effective) *df* used.

For the nonparametric model, smoothing splines calculated by generalized additive models were fitted to examine the unbiased shape of the potential learning curves, exhibiting different effective *df* per test [25,26].

For the parametric models, simple linear (*df*=4) and linear quadratic (*df*=5) mixed models were fitted, both using time and in addition the latter using time squared as fixed effects. As the third parametric model, we considered bounded growth mixed models (*df*=6) using the following formula:

y_(_*_t_*_)_ = boundary + (y_0_ - boundary) *e^-ct^*
**(1)**

We treated boundary and baseline (y_0_) as random effects, while we considered the growth constant *c* as a fixed effect.

#### Sensitivity Analyses

In addition to our main analysis on practice effects as a function of repetition with the selection criteria of a minimum of 5 weeks and 5 repetitions, we performed 3 additional sensitivity analyses: sensitivity analyses 1 and 3 were modeling practice effects as a function of weeks since the first test instead of the number of repetitions, and sensitivity analyses 2 and 3 were performed using stricter selection criteria of a minimum of 10 weeks and 10 repetitions (Table 1).

**Table 1:** Comparison of the main analysis with the 3 sensitivity analyses performed.

Criteria	Minimum of 5 weeks and 5 repetitions	Minimum of 10 weeks and 10 repetitions
Practice effects as a function of number of repetitions	Main analysis	Sensitivity analysis 2
Practice effects as a function of weeks since first test	Sensitivity analysis 1	Sensitivity analysis 3

All statistical analyses were performed using R 4.0.3 (R Foundation for Statistical Computing). Point estimates are accompanied by 95% CI in brackets, unless otherwise stated. *P* values were based on two-tailed *t* tests, unadjusted unless otherwise stated and considered significant if <.05. All analysis codes can be found on the web [27]. The data set used can be found on the web [28].

## Results

### Overview

Of the 1147 patients who performed at least one cognitive *e-SDMT*, 262 (22.8%) fulfilled our selection criteria of a minimum of 5 repetitions and 5 weeks between the first and last repetitions, accounting for 77.31% (4824/6240) of all performed *e-SDMT* tests. For *Finger Pinching* and *Draw a Shape*, 23.8% (264/1109) and 24% (259/1079) patients were selected, accounting for 87.14% (19,650/22,550) and 87.18% (19,019/21,816) of the performed tests, respectively. For *Two Minute Walk*, *U-Turn,* and *Static Balance*, 29.7% (171/575), 24.1% (217/901), and 24.34% (257/1056) patients were selected, representing 92.79% (14,393/15,512), 89.37% (15,051/16,841), and 88.41% (16,797/19,000) of the respective tests (Table 2). The minimum intertest interval was constrained by the app to 7 days for *e-SDMT*, 2 days for *Finger Pinching* and *Draw a Shape* for each hand, and 1 day for *Two Minute Walk*, *U-Turn, and Static Balance*, explaining the lower number of *e*-*SDMT* repetitions. However, participants had highly variable intertest intervals, making this an irregular time series, as indicated by each participant’s median intertest interval and IQR. Table 2 shows the median of these statistics for all the selected participants.

**Table 2:** Characteristics of included patients with multiple sclerosis.

Domain		Cognition	Dexterity			Mobility		
		*Electronic Symbol Digit Modalities Test*	*Finger Pinching*	*Draw a Shape*	*Two Minute Walk*		*U-Turn*	*Static Balance*
**Number of patients meeting selection criteria**								
	Total, N	1147	1109^a^	1079^b^	575		901	1056
	Selected, n (%)	262 (22.8)	264 (23.8)	259 (24)	171 (29.7)		217 (24.1)	257 (24.3)
**Number of tests performed by these patients**								
	Total, N	6240	22,550	21,816	15,512		16,841	19,000
	Selected, n (%)	4824 (77.3)	19,650 (87.1)	19,019 (87.2)	14,393 (92.8)		15,051 (89.4)	16,797 (88.4)
**Sex**								
	Total, N	262	499	484	171		217	257
	Female, n (%)	184 (70.2)	353 (70.7)	345 (71.3)	123 (71.9)		155 (71.4)	181 (70.4)
Age (years), median (IQR; range)		50.2 (42.0-58.0; 20.0-79.0)	50.0 (41.8-58.0; 20.0-79.0)	49.6 (41.5-58.0; 20.0-79.0)	50.0 (41.6-58.1; 20.0-74.5)		49.6 (41.5-57.0; 20.0-79.0)	48.7 (41.1-57.0; 20.0-79.0)
Number of repetitions, median (IQR; range)		11 (7-18; 5-119)	17 (9-41.5; 5-416)	17 (9-41; 5-414)	30 (15-83.5; 5-827)		24 (11-69; 5-829)	24 (12-67; 5-828)
Median of intertest intervals (days), median (IQR; range)		7.9 (7.1-10.2; 6.7-87.1)	3.1 (2.1-5.4; 1.9-42.8)	3.3 (2.2-5.6; 1.9-77.6)	1.4 (1.0-3.0; 1.0-24.9)		1.8 (1.1-3.3; 1.0-25.4)	1.7 (1.1-3.1; 0.7-28.4)
Median of IQR of intertest intervals (days), median (IQR; range)		3.6 (1.0-9.9; 0.0-133.8)	3.3 (1.1-8.0; 0.0-198.1)	3.4 (1.1-8.8; 0.0-251.0)	2.0 (0.9-5.1; 0.1-39.2)		2.6 (0.9-6.9; 0.0-81.5)	2.3 (0.8-7.7; 0.0-93.0)
Number of weeks from the first to the last test, median (IQR; range)		18.3 (11.5-54.1; 5.0-164.7)	17.4 (10.9-49.4; 5.0-164.7)	17.9 (11.0-49.9; 5.0-164.7)	17.2 (11.1-48.2; 5.0-146.3)		16.3 (10.3-47.0; 5.0-146.3)	16.7 (10.3-47.0; 5.0-152.1)

^a^With 26.19% (499/1905) of hands selected.

^b^With 26.23% (484/1845) of hands selected.

### Cognition: e-SDMT

**Figure 1:**
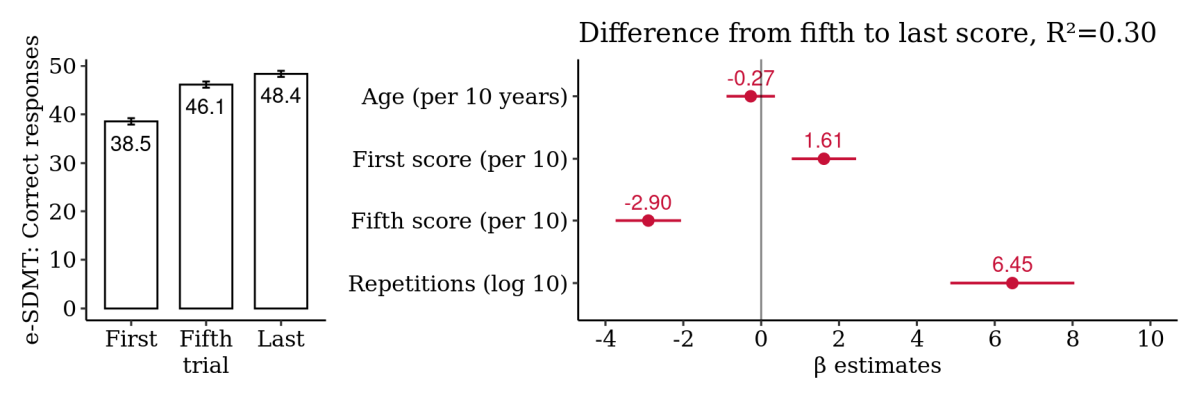
Patient-level summary analysis for the *electronic Symbol Digit Modalities Test*: comparison of the first, fifth, and last score. Multivariate association of the difference from the fifth to last score with age, first and fifth score, and the log-transformed number of repetitions (n=262 patients). *e-SDMT*: *electronic Symbol Digit Modalities Test*.

A summary analysis of the 262 selected patients yielded a mean difference from the first to last score of 9.8 correct responses, representing an average observed improvement of 25.4% (95% CI 23.1% to 27.8%) from the first score. Although the majority of this improvement (19.7%, 95% CI 17.5% to 21.9%) occurred up to the fifth score and can thus be considered a short-term learning effect, there was still a significant improvement from the fifth score onward of, on average, 5.7% (95% CI 4.1% to 7.4%), suggesting a long-term practice effect. A multivariate regression model of this difference yielded a significant association with the total number of repetitions, further supporting the long-term practice effects (Figure 1). Age was positively correlated with the number of repetitions performed (Pearson correlation coefficient, *R*=0.19; *P*=.003), but the first score was not (*R*=−0.03; *P*=.70; Multimedia Appendix 1).

**Figure 2:**
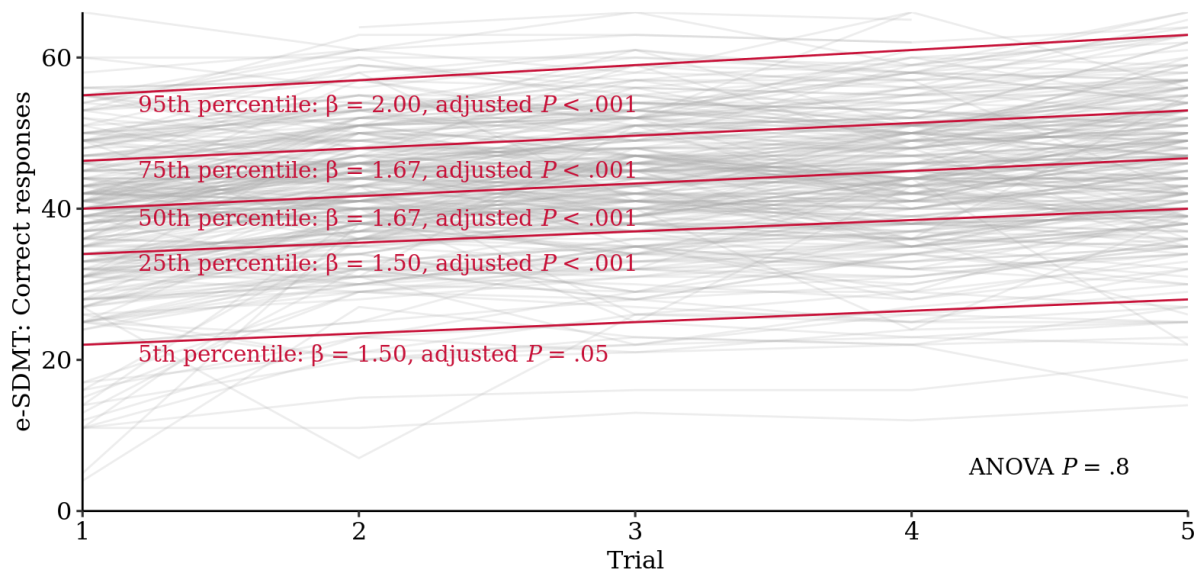
Linear quantile regression for the *electronic Symbol Digit Modalities Test* of short-term learning effects up to the fifth repetition. Comparison of baseline performance and linear slope of low (5th and 25th percentiles), median, and high performers (75th and 95th percentiles). Quantile regression *P* values are Bonferroni-adjusted (n=1310 tests). ANOVA: analysis of variance; *e-SDMT*: *electronic Symbol Digit Modalities Test*.

When comparing performances by 5th, 25th, 50th, 75th, and 95th percentile groups up to the fifth trial with quantile regression, baseline performances were normally distributed with intercept estimates of 22.0 (19.1-24.9), 34.0 (32.7-35.3), 40.0 (38.8-41.2), 46.3 (45.1-47.6), and 55.0 (53.4-56.6), respectively. The ANOVA-type test for all 5 slopes (β=1.5-2.0) did not suggest that short-term learning rates for these groups differed significantly (*P*=.80; Figure 2).

**Figure 3:**
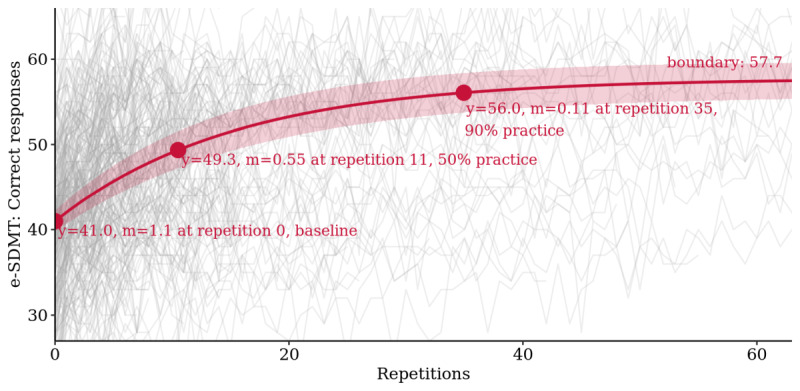
Learning curve analysis for the *electronic Symbol Digit Modalities Test*: bounded growth mixed model of practice effects with 95% CI band and baseline, 50% and 90% practice points marked (m=slope of tangent; n=4824 tests). *e-SDMT*: *electronic Symbol Digit Modalities Test*.

The long-term learning curve analysis showed that the bounded growth model fit the data best with an RMSE of 3.3 correct responses, followed by 3.6 for the smoothing spline, 3.8 for the quadratic, and 4.0 for the linear model (Multimedia Appendix 2). Strong boundary practice effects were found with baseline estimates of on average 41.0 (95% CI 39.8 to 42.2) correct responses and boundary estimates of 57.7 (95% CI 55.7 to 59.8) correct responses, leading to an average improvement over baseline of 40.8% (95% CI 39.9% to 41.6%). Half of the practice effect was reached after 11 repetitions and 90% after 35 repetitions (Figure 3).

For *e-SDMT* corrected for dexterity and reaction speed, Spearman correlation of all 6190 corrected scores with their uncorrected counterpart yielded ρ=0.55. The resulting practice effects were very similar to the uncorrected *e-SDMT*, with an observed improvement from the first to last score of 19% (95% CI 16.1% to 22%), consisting of 12.4% (95% CI 9.8% to 15%) from the first to fifth score, and 6.6% (95% CI 4.6% to 8.7%) from the fifth to last score. As with the uncorrected *e-SDMT*, no significant differences in short-term learning rates were found between low, median, and high performers (ANOVA-type *P*=.30). Boundary long-term improvements were 23.5% (95% CI 23% to 24%), with half of the practice effect reached after 7 repetitions and 90% practice effect reached after 23 repetitions (Multimedia Appendices 3-6).

### Dexterity: Finger Pinching

**Figure 4:**
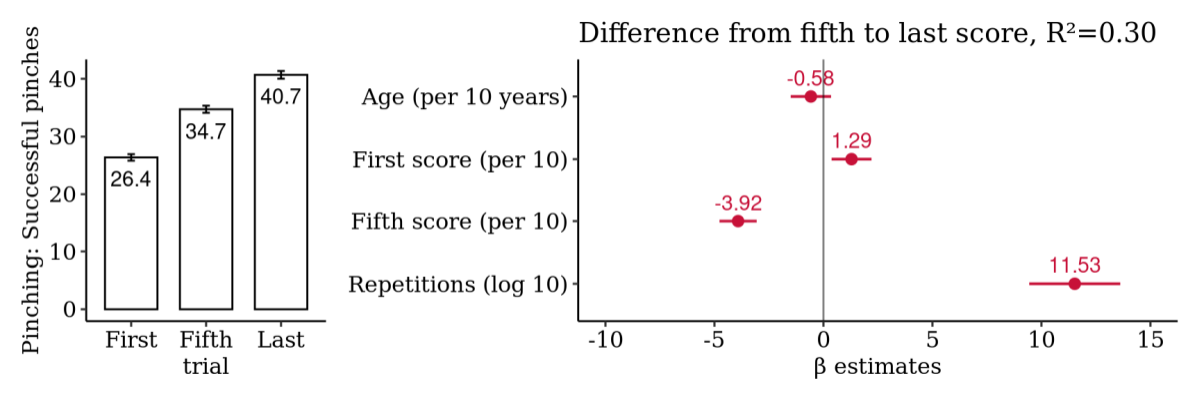
Hand-level summary analysis for *Finger Pinching*: comparison of the first, fifth, and last score. Multivariate association of the difference from the fifth to last score with age, first and fifth score, and the log-transformed number of repetitions (n=499 hands).

A summary analysis of the 499 selected hands yielded a mean difference from the first to last score of 14.3 successful pinches, representing an average observed improvement of 54.2% (95% CI 49.3% to 59.1%) over the first score. Similar to the findings on the *e-SDMT*, the majority of this improvement (31.5%, 95% CI 27.5% to 35.4%) occurred up to the fifth score, compatible with a short-term learning effect. However, the remaining improvement of 22.7% (95% CI 18.6% to 26.8%) occurred after the fifth trial. This improvement was significantly associated with the total number of repetitions, indicating a strong long-term practice effect (Figure 4). Age was positively correlated with the number of repetitions performed (*R*=0.21; *P*<.001) but the first score was not (*R*=−0.06; *P*=.20; Multimedia Appendix 7).

**Figure 5:**
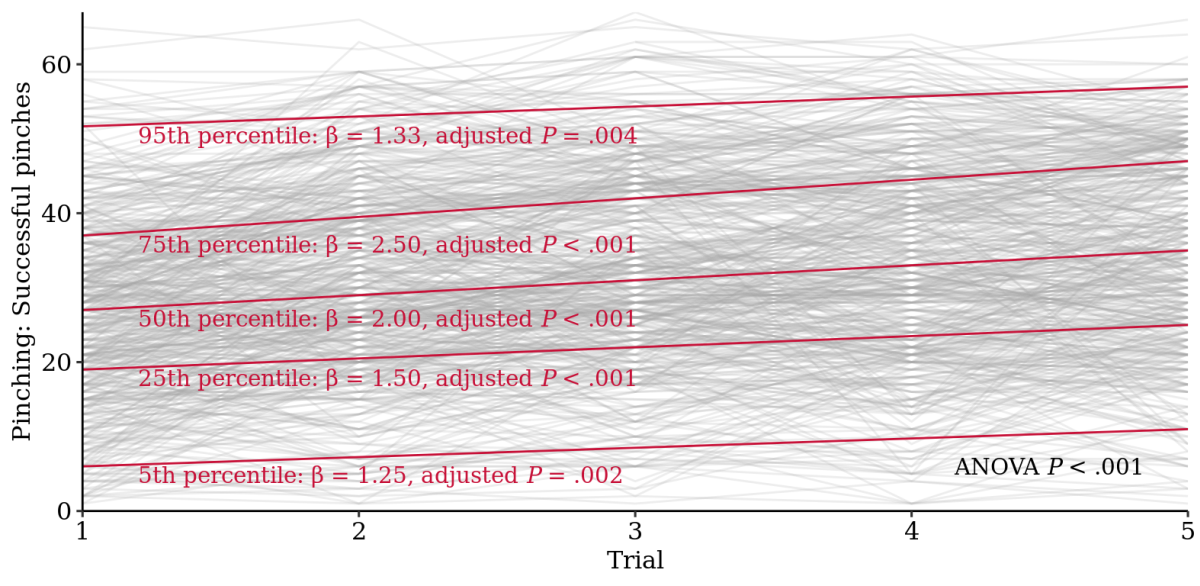
Linear quantile regression for *Finger Pinching* of short-term learning effects up to the fifth repetition. Comparison of baseline performance and linear slope of low (5th and 25th percentiles), median, and high performers (75th and 95th percentiles). Quantile regression *P* values are Bonferroni-adjusted (n=2495 tests). ANOVA: analysis of variance.

Baseline performances were normally distributed with intercept estimates of 6.0 (95% CI 4.5 to 7.5) for the fifth percentile, 19.0 (95% CI 17.8 to 20.2) for the 25th, 27.0 (95% CI 25.8 to 28.2) for median performers, 37.0 (95% CI 35.6 to 38.4) for the 75th, and 51.7 (95% CI 49.6 to 53.8) for the 95th percentile with quantile regression. The β coefficients for short-term learning up to the fifth trial were the highest for the 75th percentile and median performers with 2.50 (95% CI 1.96 to 3.04) and 2.00 (95% CI 1.45 to 2.55) additional successful pinches per repetition, lower for the 25th percentile (1.50, 95% CI 1.00 to 2.00) and the lowest for the 5th and 95th percentiles (1.25, 95% CI 0.57 to 1.93, and 1.33, 95% CI 0.56 to 2.11, respectively). These differences in slopes between performance levels were significant (ANOVA-type *P*<.001; Figure 5).

**Figure 6:**
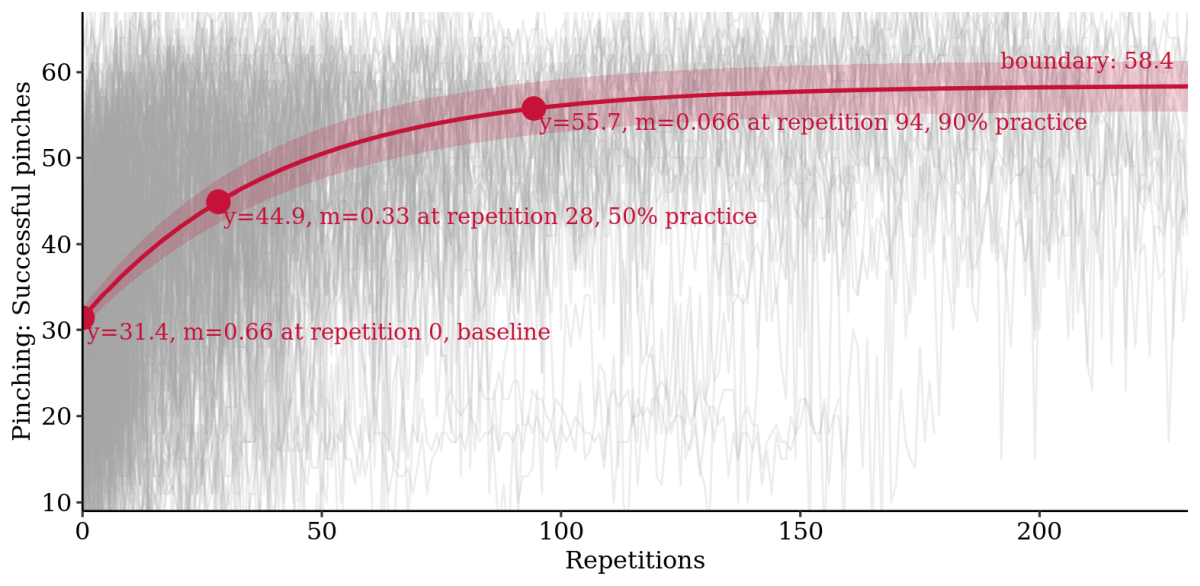
Learning curve analysis for *Finger Pinching*: bounded growth mixed model of practice effects with 95% CI band and baseline, 50%, and 90% practice points marked (m=slope of tangent; n=19,650 tests).

Long-term learning curve analysis again showed that the bounded growth model fit the data best with an RMSE of 6.8 successful pinches, followed by 7.5 for the smoothing spline, 7.9 for the quadratic, and 8.1 for the linear model (Multimedia Appendix 8). Strong boundary practice effects were found with baseline estimates of, on average, 31.4 (95% CI 30.2 to 32.5) and boundary estimates of 58.4 (95% CI 55.5 to 61.4) successful pinches, leading to an average improvement over baseline of 86.2% (95% CI 83.6% to 88.7%). Half of the practice effect was reached after 28 repetitions and 90% after 94 repetitions (Figure 6).

### Dexterity: Draw a Shape

**Figure 7:**
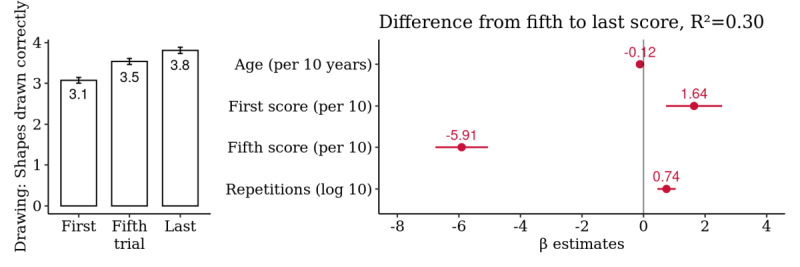
Hand-level summary analysis for *Draw a Shape*: comparison of the first, fifth, and last score. Multivariate association of the difference from the fifth to the last score with age, first and fifth score, and the log-transformed number of repetitions (n=484 hands).

A summary analysis of the 484 selected hands yielded a mean improvement in the number of shapes drawn correctly from the first to last score of 23.9% (95% CI 18.3% to 29.5%), from the first to fifth score of 15.1% (95% CI 9.8% to 20.3%), and from the fifth to last score of 8.8% (95% CI 3.8% to 13.8%). This difference was significantly associated with the total number of repetitions, suggesting a long-term practice effect (Figure 7). Age was positively correlated with the number of repetitions performed (*R*=0.22; *P*<.001) but the first score was not (*R*=−0.08; *P*=.09; Multimedia Appendix 9).

Intercept estimates for baseline performances were 1 shape drawn correctly for the 5th percentile, 2 for the 25th percentile, 3 for the median performers, 5 for the 75th percentile, and 6 for the 95th percentile with quantile regression. In this analysis, only median performers showed a significant short-term learning rate up to the fifth trial (Multimedia Appendix 10).

**Figure 8:**
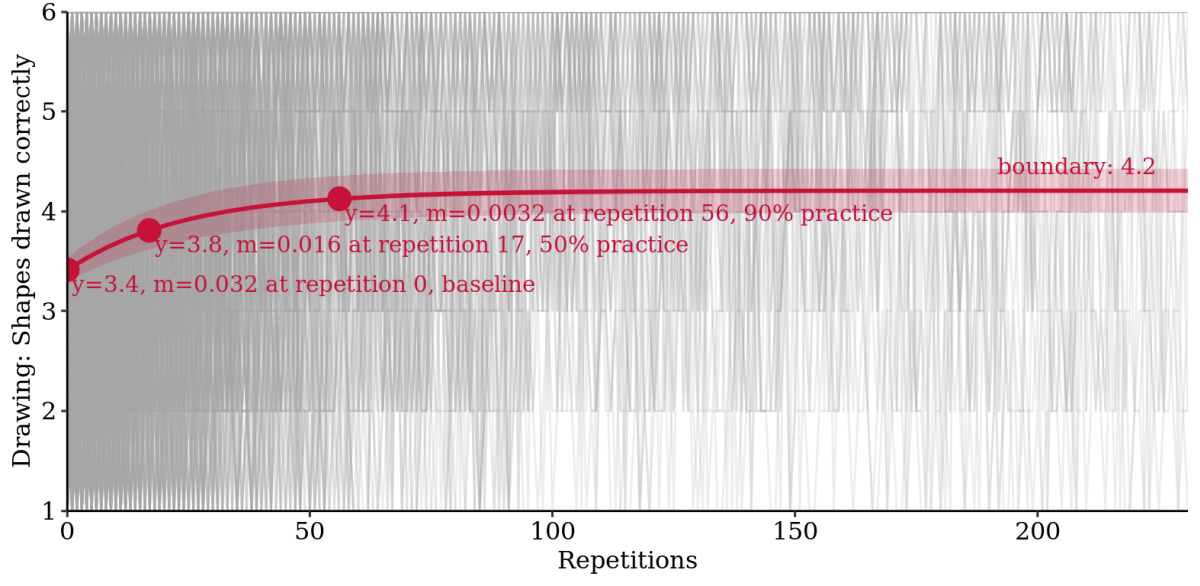
Learning curve analysis for *Draw a Shape*: bounded growth mixed model of practice effects with 95% CI band and baseline, 50%, and 90% practice points marked (m=slope of tangent; n=19,019 tests).

The long-term learning curve analysis again showed that bounded growth models fit the data best with an RMSE of 1.02 shape drawn correctly, followed by 1.06 for the smoothing spline, 1.07 for the quadratic, and 1.08 for the linear model (Multimedia Appendix 11). Boundary practice effects were found with an average improvement over baseline of 23.1% (95% CI 20.9% to 25.2%), reaching half of the practice effect after 17 repetitions and 90% after 56 repetitions (Figure 8).

### Mobility: Two Minute Walk

**Figure 9:**
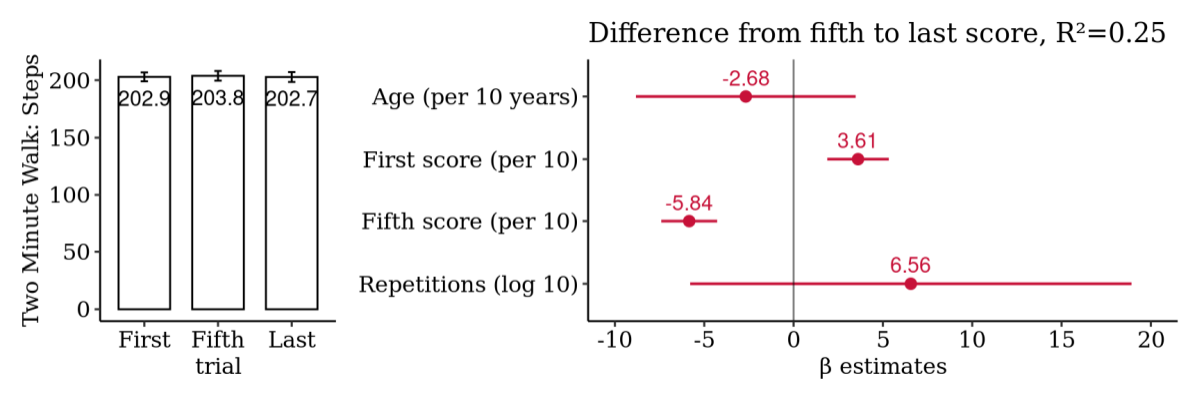
Patient-level summary analysis for *Two Minute Walk*: comparison of the first, fifth, and last score. Multivariate association of the difference from the fifth to the last score with age, first and fifth score, and the log-transformed number of repetitions (n=171 patients).

A summary analysis of the 171 selected patients yielded no significant difference between the first, fifth, and last scores with a mean difference from the fifth to last score of 1.4 (95% CI −5.2 to 7.9) steps. This difference was also not associated with the total number of repetitions performed (Figure 9 and Multimedia Appendix 12).

The distribution of baseline performance was left-skewed with performers in the 5th percentile achieving, on average, 87.0 (95% CI 52.2 to 121.8) steps; in the 25th percentile, 181.0 (95% CI 170.7 to 191.3) steps; on median, 219.0 (95% CI 212.6 to 225.4) steps; in the 75th percentile, 236.0 (95% CI 230.6 to 241.4) steps; and in the 95th percentile, 260.0 (95% CI 255.2 to 264.8) steps. No significant slopes up to the fifth trial could be observed (Multimedia Appendix 13).

### Mobility: U-Turn

**Figure 10:**
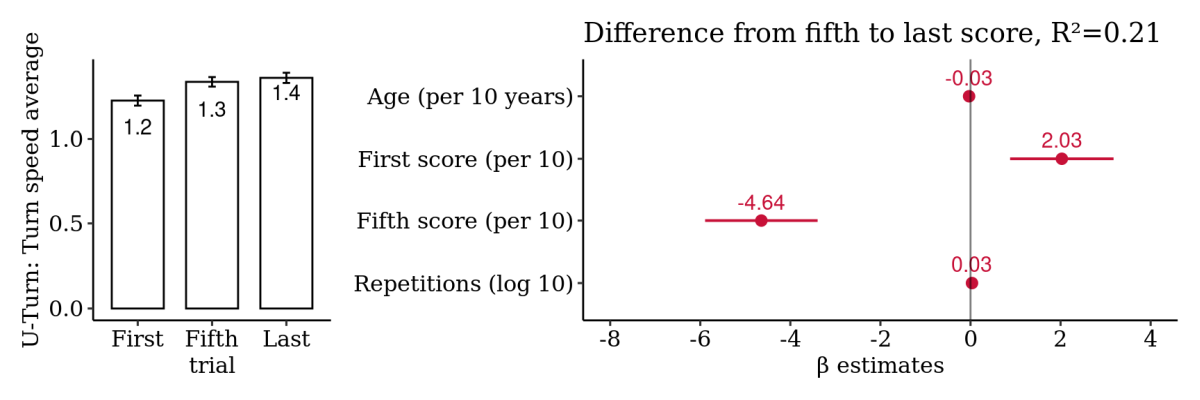
Patient-level summary analysis for *U-Turn*: comparison of the first, fifth, and last score. Multivariate association of the difference from the fifth to the last score with age, first and fifth score, and the log-transformed number of repetitions (n=217 patients).

A summary analysis of the 217 selected patients yielded a significant improvement from the first to last score with a mean difference in turn speed average of 0.13 rad/s, representing an average observed difference of 11.0% (95% CI 5.7% to 16.2%) over the first score. However, the majority of this difference occurred up to the fifth score (9%, 95% CI 3.7% to 14.3%), and the remaining difference from the fifth to last score (1.9%, 95% CI −2.3% to 6.1%) was neither significant nor associated with the total number of repetitions performed (Figure 10 and Multimedia Appendix 14).

Baseline performances estimated with quantile regression were normally distributed with 0.5 rad/s (95% CI 0.5 to 0.6) for the 5th percentile, 0.9 rad/s (95% CI 0.9 to 1.0) for the 25th percentile, 1.3 rad/s (95% CI 1.2 to 1.3) for median performers, 1.5 rad/s (95% CI 1.5 to 1.6) for the 75th percentile, and 2.0 rad/s (95% CI 1.9 to 2.1) for the 95th percentile groups. Only the slope of the 25th percentile group was significant in this analysis up to the fifth trial (β=.04; 95% CI 0.02 to 0.06), and the difference in slopes was not significant in the ANOVA-type test (*P*=.40; Multimedia Appendix 15).

### Mobility: Static Balance

**Figure 11:**
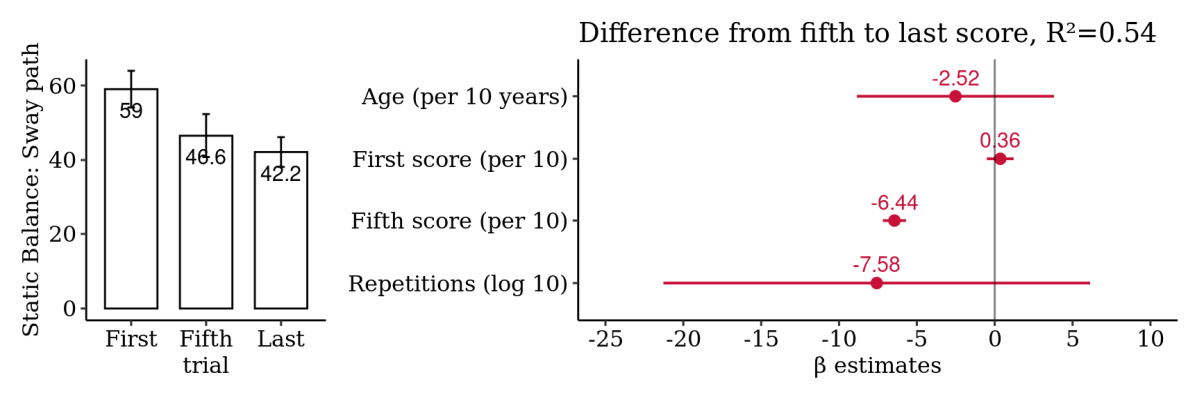
Patient-level summary analysis for *Static Balance*: comparison of the first, fifth, and last score. Multivariate association of the difference from the fifth to the last score with age, first and fifth score, and the log-transformed number of repetitions (n=257 patients).

A summary analysis of the 257 selected patients yielded a significant difference from the first to last score, with a mean difference in sway path of −16.9 m/s². This is the only test in which fewer numbers are better. Thus, the average observed improvement was −28.6% (95% CI −48.6% to −8.5%) over the first score. However, the majority of this improvement occurred up to the fifth score (−21.1%, 95% CI −45% to −2.8%), and the remaining difference from the fifth to last score (−7.5%, 95% CI −24.1% to 9.2%) was neither significant nor associated with the total number of repetitions performed (Figure 11 and Multimedia Appendix 16).

Baseline performance estimates were strongly right-skewed with 5.7 m/s² (95% CI 4.3 to 7.0) for the 5th percentile, 11.7 m/s² (95% CI 9.4 to 14.0) for the 25th percentile, 23.8 m/s² (95% CI 20.7 to 26.8) for the median performers, 68.0 m/s² (95% CI 55.7 to 80.3) for the 75th percentile, and 260.2 m/s² (95% CI 200.4 to 320.0) for the 95th percentile. In this test, the 5th and 25th percentiles are the top performing groups, and their quantile regression slopes up to the fifth trial are not significant. However, the significant negative slopes of median (β=−2.32; 95% CI −3.47 to −1.17), 75th percentile (β=−8.46; 95% CI −12.21 to −4.72), and 95th percentile (β=−27.25; 95% CI −46.30 to −8.20) performers were increasingly steep, and the overall ANOVA-type difference test yielded *P*<.001(Multimedia Appendix 17).

### Sensitivity Analyses

The results of the sensitivity analyses were in line with the results of the main analysis. Sensitivity analysis 2, which used stricter inclusion criteria with a minimum of 10 weeks and 10 repetitions, was overall very similar with expected further increases in mean improvement from the fifth to last score (mean improvement 5.7% for main analysis vs 9.1% for sensitivity analysis 2 for *e-SDMT*; 22.7% for main analysis vs 35.0% for sensitivity analysis 2 for *Finger Pinching*; and 8.8% for main analysis vs 13.3% for sensitivity analysis 2 for *Draw a Shape*) and a slight decrease in average boundary increase in performance (average boundary increase 40.8% for main analysis vs 34.3% for sensitivity analysis 2 for *e-SDMT*; 86.2% for main analysis vs 73.8% for sensitivity analysis 2 for *Finger Pinching*; and 23.1% for main analysis vs 19.8% for sensitivity analysis 2 for *Draw a Shape*). Sensitivity analyses 1 and 3, which modeled practice effects as a function of weeks since the first test instead of the number of repetitions, also supported the main findings. However, the association of the maximum number of weeks from the first to the last tests with the difference from the fifth to last score was generally lower and so was the average increase in performance (Multimedia Appendix 18).

## Discussion

### Principal Findings

Strong long-term practice effects were found for *e-SDMT*, *Finger Pinching*, and *Draw a Shape*, with mean observed improvements of 25.4%, 54.2%, and 23.9% from the first to last score, respectively. Of these, 5.7%, 22.7%, and 8.8% occurred from the fifth score onward. However, the number of repetitions differed widely among participants with a range of 5-119 repetitions for *e-SDMT* (median 11), 5-416 for *Finger Pinching* (median 17), and 5-414 for *Draw a Shape* (median 17), introducing bias. To estimate boundary practice effects independent of the number of repetitions in our sample, we modeled learning curves with bounded growth models, a subtype of nonlinear mixed models. This approach yielded boundary improvements over the baseline of 40.8% for *e-SDMT*, 86.2% for *Finger Pinching*, and 23.1% for *Draw a Shape*. Interestingly, the practice effect seemed to last longer for the dexterity tests *Finger Pinching* and *Draw a Shape*, reaching half of the practice effect after repetition 28 and 17, respectively, compared to repetition 11 for *e-SDMT*.

These practice effects likely include both short-term learning effects, where patients become acquainted with the tests, and long-term practice effects. We believe these effects have not only different origins, time scales, and magnitudes but also different implications for the use of digital assessments in clinical studies and clinical practice. Short-term learning effects can be addressed by ensuring that participants have sufficient training before the observational period; long-term practice effects constitute a significant challenge for all applications beyond trials with a comparator arm. Although these effects are impossible to untangle in an unsupervised setting like this, we considered improvements up to the fifth trial to be more likely due to short-term learning and improvements afterward more likely because of long-term practice effects, based on the recommendation to use the fifth trial of the 9HPT as baseline [24].

For *U-Turn* and *Static Balance*, only short-term learning effects could be observed, ceasing after a maximum of 5 repetitions. Interestingly, for *Static Balance*, these short-term learning effects were not present in those with high baseline performance and were most pronounced in those with low baseline performance, potentially highlighting that the test instructions were not clear from the beginning. For *Two Minute Walk*, neither short-term learning nor long-term practice effects were observed.

For *e-SDMT*, quantile regression analysis suggested that the short-term learning rate was independent of the baseline performance. However, for *Finger Pinching*, median and high performers improved significantly faster than low and highest performers, with the learning rate decreasing toward the extremes. One can hypothesize that low performers might be more physically disabled, preventing them from improving as quickly as the median performers. On the other hand, the highest performers might reach their boundary sooner, leaving less room for improvement.

The 3 sensitivity analyses confirmed our main findings. However, for sensitivity analyses 1 and 3, which modeled practice effects as a function of weeks since the first test instead of the number of repetitions, the effect sizes were smaller. We believe this is caused by the irregular nature of these time-series data, as the intertest intervals differed widely, highlighting a complication in user-scheduled testing (Table 2).

### Comparison With Previous Work

#### Overview

Only a few studies have examined practice effects in smartphone-based tests for patients with MS. Bove et al [14] analyzed the data from 38 patients, 22 of whom completed the planned study period of 12 months. They found strong practice effects for both their custom-made cognitive tests (digital adaptations of the trail-making test, the n-back test, a verbal fluency test, and an attention test), and a digital adaptation of the 9HPT. Interestingly, they suggest using person-specific learning curves quantified by binary spline inflection point analysis as a potential outcome measure [14].

In addition, Liao et al [29] recently reported significant practice effects for information processing speed and manual dexterity but not for walking speed in a tablet-based test battery called *MS Performance Test*, broadly confirming our results. However, they only analyzed 2-5 repetitions per patient and per test and could thus not examine long-term practice effects. Interestingly, they found that younger age was associated with larger practice effects, whereas we observed the opposite for *e-SDMT* and *Finger Pinching* (Multimedia Appendices 1 and 7), potentially highlighting differences between their low-frequency and our high-frequency testing.

#### Cognition: SDMT

Practice effects are well known for SDMT in both healthy controls and patients with MS, although the effect sizes reported were highly variable. Morrow et al [30] studied 660 natalizumab-treated patients with MS with a total of 13 repetitions of the oral SDMT over 48 weeks with average baseline scores of 46.8 (SD 15.3) correct responses and average final scores of 62.2 (SD 18.1) at week 48, resulting in an average improvement of 32.9% over baseline. Although the improvement was most pronounced over the first 3 repetitions, there was no obvious boundary [30].

In contrast, Benedict et al [31] found only minimal practice effects in 76 patients with MS with a total of 6 repetitions of oral SDMT over 5 months. Average baseline scores of 49.8 (SD 12.4) correct responses and average final scores of 52.5 (SD 14.3) at month 5, representing an improvement of only 5.4% over baseline, were found. However, their 25 healthy controls improved from 62.0 (SD 11.3) to 71.4 (SD 13.2), representing a practice effect of 15.1% [31].

Roar et al [32] examined practice effects in 80 natalizumab-treated patients with MS with up to 31 repetitions over 30 months and reported improvements of roughly 25% over baseline, on average, with the rate of improvement slowing down after 6 months. Baseline performance and relative improvement were worse for the more severely affected patients with MS [32]. Interestingly, rearrangement of the SDMT symbol key resulted in a return to baseline performance, suggesting that the practice effect could be attributed to key memorization and that no generalizable learning or improvement of processing speed occurred [32].

Indeed, all of the traditional paper and pencil SDMT versions have the limitation of a fixed key, which is why Benedict et al [33] recommend the use of equivalent forms with alternate keys to mitigate practice effects. The smartphone-based *e-SDMT* version examined in this study has changing keys, thus emulating the process of alternate forms. Key changes are not truly random, and the subtlety of the original SDMT, in which the first 26 items only use the first 6 symbols in the key, is preserved [34].

With our result of an average boundary improvement over baseline of 40.8%, we can show that with weekly testing, practice effects for SDMT are likely to be stronger than with monthly testing, as performed by the abovementioned studies, and at least partly independent of the key.

As a limitation, the smartphone-based test in this study was not oral but based on touching a number pad, potentially biasing the results by dexterity problems (and dexterous practice effects). However, our analysis of *e-SDMT* corrected for dexterity and reaction speed by using Floodlight’s *baseline* showed very similar results, suggesting that this is not a major issue, potentially because patients are free to use their preferred hand for this test.

#### Dexterity: 9HPT

Practice effects often become apparent in the examination of test-retest, intrarater, and interrater reliability. In this way, Cohen et al [35] found evident practice effects for the 9HPT first in 10 and later in 436 secondary progressive patients with MS over 4-6 repetitions in up to 4 weeks, which stabilized after 3 repetitions [21].

Solari et al [24] found even stronger practice effects in 32 patients with MS with 6 repetitions in 24 hours, which stabilized after 4 repetitions. As a consequence, they recommend performing 9HPT 4 times before baseline in any study to mitigate practice effects [24].

The smartphone-based *Finger Pinching* and, to a slightly lesser extent, also the *Draw a Shape* tests we examined seem to have much stronger and longer-lasting practice effects than the 9HPT. It can be speculated that high-frequency testing (ie, sustained daily practice over multiple months) maximizes the long-term practice effect.

#### Mobility: T25FW

No practice effects were found for T25FW, which was examined alongside 9HPT in the abovementioned studies [21,24,35]. This result is in line with our finding of no long-term practice effect in the smartphone-based mobility tests, *Two Minute Walk* and *U-Turn*, which have been validated with the T25FW [9]. However, the outcome reported for *Two Minute Walk* in this study (ie, number of steps) is a quantitative gait parameter and thus representative of endurance, unlike the more qualitative gait parameter *step power* used by Montalban et al [9] for validation against T25FW.

### Limitations

MS diagnoses of study participants were self-declared, and there was no confirmation or assessment by health professionals. In addition, no clinical information was available for the participants to compare with their performance in digital tests. Differences caused by disease duration, severity, or treatment could not be analyzed.

In addition, we observed a high variability of results, which is most likely partly due to biomedical day-to-day fluctuations and partly due to circumstantial and technical noise, for example, caused by interrupted test performance or sensor error. However, it is impossible to determine these effects using the present data set.

Finally, these time-series data are highly irregular and have strong right-skewness. Our models expect data missing at random. We found no evidence that baseline performance influenced adherence and the number of repetitions, but age was found to be a confounder for all domains. Interestingly, older people tended to perform more repetitions than younger people (*R*=0.19 for *e-SDMT*; *R*=0.21 for *Finger Pinching*; and *R*=0.22 for *Draw a Shape*; Multimedia Appendices 1, 7, and 9). One can hypothesize that this is because older patients with MS tend to be more severely affected and thus might have higher intrinsic motivation. Another possible explanation is that younger people might have more competing time commitments, for example, because of their occupation or family. As age was associated with larger observed improvements from the fifth to last score for *e-SDMT* and *Finger Pinching* (both *R*=0.16; Multimedia Appendices 1 and 7), this confounder might lead to slight overestimation of the practice effects. However, in the multivariate models, age was not a significant confounder (Figures 1 and 4). Nevertheless, there might be unobserved confounders that differ between those participants who quit early and those who stayed engaged for a long time, which we aimed to mitigate by sensitivity analyses 2 and 3.

### Conclusions

In summary, we analyzed the practice effects in 6 active smartphone-based tests for cognition, dexterity, and mobility performed at high frequencies. Smartphone-based tests promise to help monitor MS disease trajectories, and there are currently multiple initiatives in development [22,36-39]. Our results suggest that strong long-term practice effects in cognitive and dexterity tests must be accounted for to identify possible disease-related changes in these domains, lasting for more than 35 repetitions for *e-SDMT*, 94 for *Finger Pinching*, and 56 for *Draw a Shape*. This is important for the interpretation of these tests in the context of personalized health and in studies with no comparator arm. On the other hand, the lack of long-term practice effects in mobility tests simplifies their interpretation, even though short-term learning effects might have to be considered.

## Appendix

Multimedia Appendix 1 Pairwise associations for the <italic>electronic Symbol Digit Modalities Test</italic> of the mean age, first score, fifth score, log-transformed number of repetitions performed, last score, and the difference from the fifth to the last score and their respective histograms (n=262 patients).

Multimedia Appendix 2 Model selection for <italic>electronic Symbol Digit Modalities Test</italic>: Comparison of 4 mixed models of increasing complexity (parametric linear, quadratic and bounded growth models and the non–parametric smoothing spline model) by root mean squared error (RMSE) and (effective) degrees of freedom (eDF) used.

Multimedia Appendix 3 Comparison of correct responses of the <italic>electronic Symbol Digit Modalities Test</italic> (x-axis) and of the <italic>electronic Symbol Digit Modalities Test</italic> divided by baseline (y-axis) to correct for dexterity and reaction speed (n=6190 tests).

Multimedia Appendix 4 Patient-level summary analysis for the <italic>electronic Symbol Digit Modalities Test</italic> corrected for dexterity and reaction speed: comparison of the first, fifth, and last score. Multivariate association of the difference from the fifth to the last score with age, first and fifth score, and the log-transformed number of repetitions (n=262 patients).

Multimedia Appendix 5 Linear quantile regression for the <italic>electronic Symbol Digit Modalities Test</italic> (corrected for dexterity and reaction speed) of short-term learning effects up to the fifth repetition. Comparison of baseline performance and linear slope of low (5th and 25th percentiles), median, and high performers (75th and 95th percentiles). Quantile regression <italic>P</italic> values are Bonferroni-adjusted (n=1310 tests).

Multimedia Appendix 6 Learning curve analysis for the <italic>electronic Symbol Digit Modalities Test</italic> corrected for dexterity and reaction speed: bounded growth mixed model of practice effects with 95% CI band and baseline, 50%, and 90% practice points marked (m=slope of tangent; n=4801 tests).

Multimedia Appendix 7 Pairwise associations for <italic>Finger Pinching</italic> of the mean age, first score, fifth score, log-transformed number of repetitions performed, last score, and the difference from the fifth to the last score and their respective histograms (n=499 hands).

Multimedia Appendix 8 Model selection for <italic>Finger Pinching</italic>: comparison of 4 mixed models of increasing complexity (parametric linear, quadratic and bounded growth models, and the nonparametric smoothing spline model) by root mean squared error and (effective) degrees of freedom used.

Multimedia Appendix 9 Pairwise associations for <italic>Draw a Shape</italic> of the mean age, first score, fifth score, log-transformed number of repetitions performed, last score, and the difference from the fifth to the last score and their respective histograms (n=484 hands).

Multimedia Appendix 10 Linear quantile regression for <italic>Draw a Shape</italic> of short-term learning effects up to the fifth repetition. Comparison of baseline performance and linear slope of low (5th and 25th percentiles), median, and high performers (75th and 95th percentiles). Quantile regression <italic>P</italic> values are Bonferroni-adjusted (n=2420 tests).

Multimedia Appendix 11 Model selection for <italic>Draw a Shape</italic>: comparison of 4 mixed models of increasing complexity (parametric linear, quadratic and bounded growth models, and the nonparametric smoothing spline model) by root mean squared error and (effective) degrees of freedom used.

Multimedia Appendix 12 Pairwise associations for <italic>Two Minute Walk</italic> of the mean age, first score, fifth score, log-transformed number of repetitions performed, last score, and the difference from the fifth to the last score and their respective histograms (n=171 patients).

Multimedia Appendix 13 Linear quantile regression for <italic>Two Minute Walk</italic> up to the fifth repetition. Comparison of baseline performance and linear slope of low (5th and 25th percentiles), median, and high performers (75th and 95th percentiles). Quantile regression <italic>P</italic> values are Bonferroni-adjusted (n=855 tests).

Multimedia Appendix 14 Pairwise associations for <italic>U-Turn</italic> of the mean age, first score, fifth score, log-transformed number of repetitions performed, last score, and the difference from the fifth to the last score and their respective histograms (n=217 patients).

Multimedia Appendix 15 Linear quantile regression for <italic>U-Turn</italic> of short-term learning effects up to the fifth repetition. Comparison of baseline performance and linear slope of low (5th and 25th percentiles), median, and high performers (75th and 95th percentiles). Quantile regression <italic>P</italic> values are Bonferroni-adjusted (n=1085 tests).

Multimedia Appendix 16 Pairwise associations for <italic>Static Balance</italic> of the mean age, first score, fifth score, log-transformed number of repetitions performed, last score, and the difference from the fifth to the last score and their respective histograms (n=257 patients).

Multimedia Appendix 17 Linear quantile regression for <italic>Static Balance</italic> of short-term learning effects up to the fifth repetition (a smaller sway path is better). Comparison of baseline performance and linear slope of low (5th and 25th percentiles), median, and high performers (75th and 95th percentiles). Quantile regression <italic>P</italic> values are Bonferroni-adjusted (n=1285 tests).

Multimedia Appendix 18 Key results for the main analysis versus sensitivity analyses 1-3 for cognition, dexterity, and mobility.

## Abbreviations

9HPT: 9-hole peg test
ANOVA: analysis of variance
*e-SDMT*: *electronic Symbol Digit Modalities Test*
MS: multiple sclerosis
RMSE: root mean squared error
SDMT: Symbol Digit Modalities Test
T25FW: timed 25-foot walk
