# Supplementary figures and images for: Practice Effects of Mobile Tests of Cognition, Dexterity, and Mobility on Patients With Multiple Sclerosis: Data Analysis of a Smartphone-Based Observational Study
Source: J Med Internet Res. 2021 Nov 18;23(11):e30394. doi: 10.2196/30394 (PMC8663564; doi:10.2196/30394)

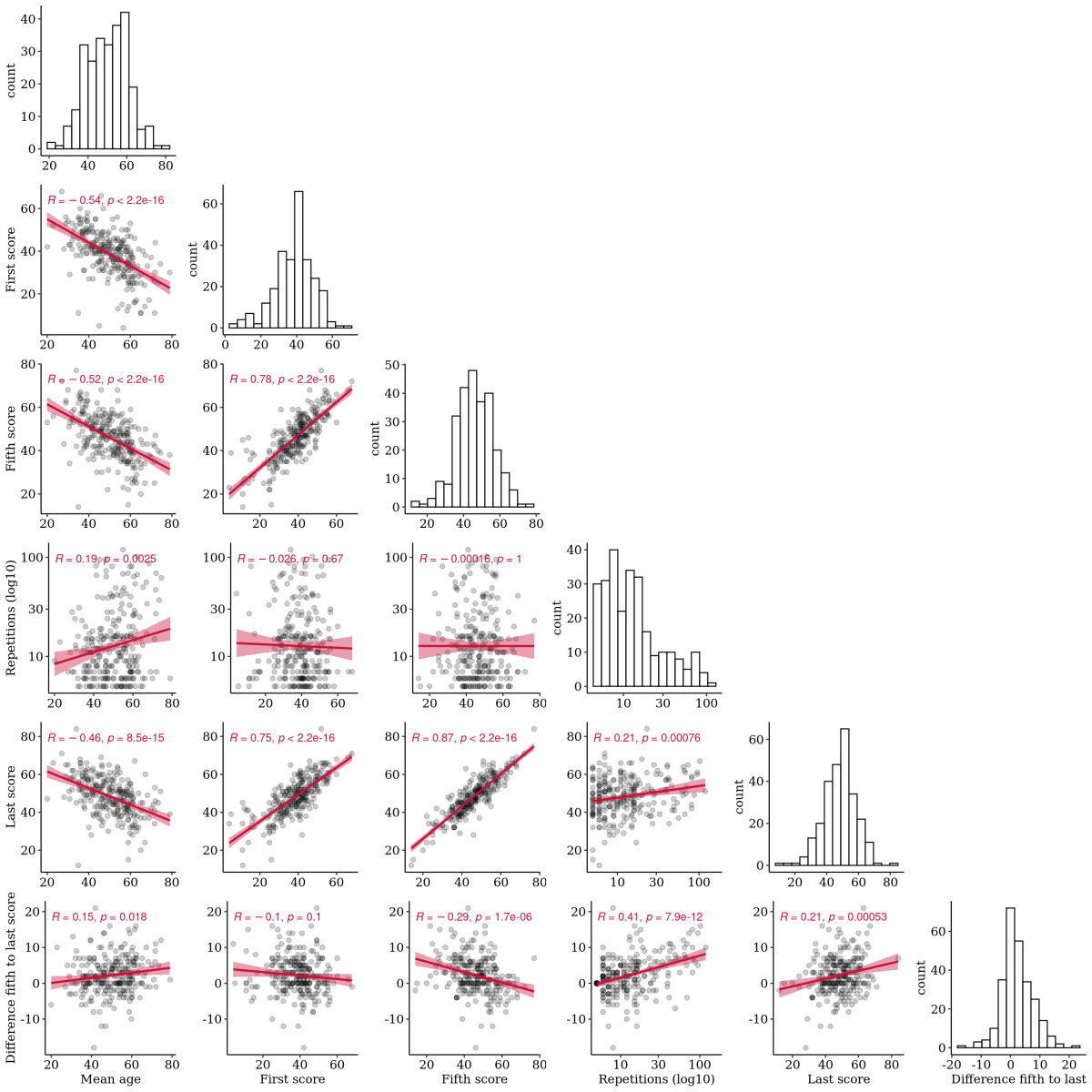

Supplement: Multimedia Appendix 1 [file jmir_v23i11e30394_app1.png]

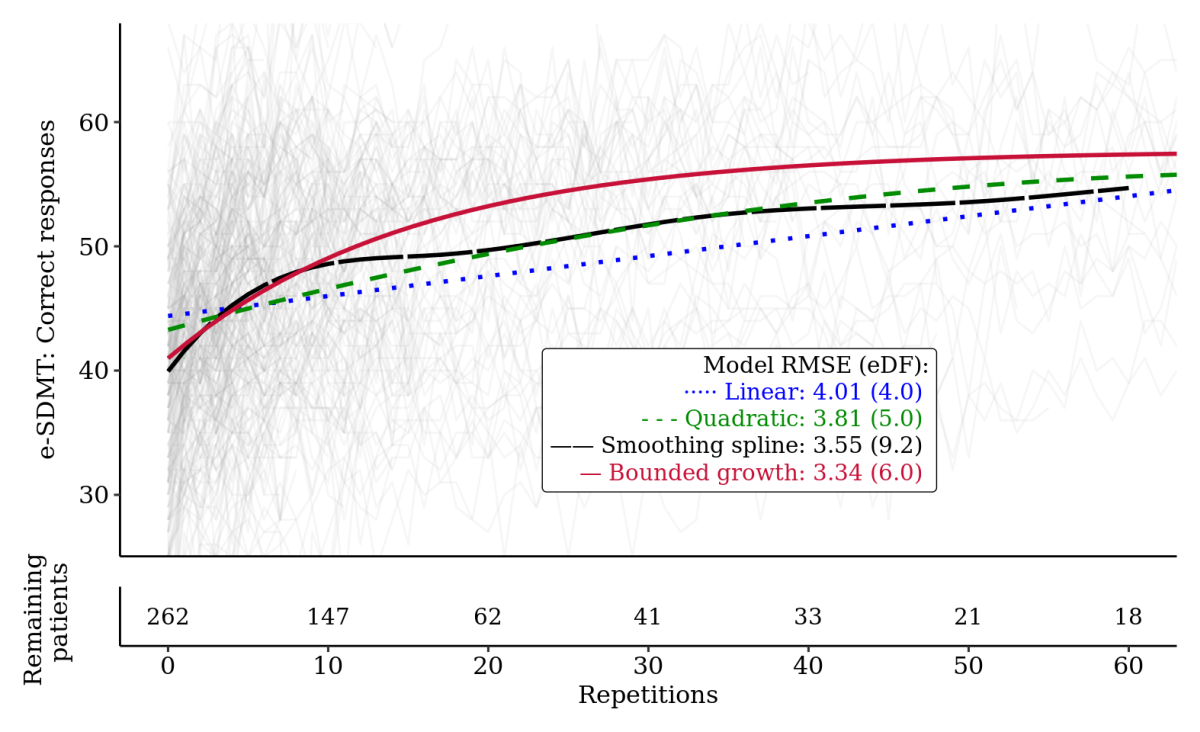

Supplement: Multimedia Appendix 2 [file jmir_v23i11e30394_app2.png]

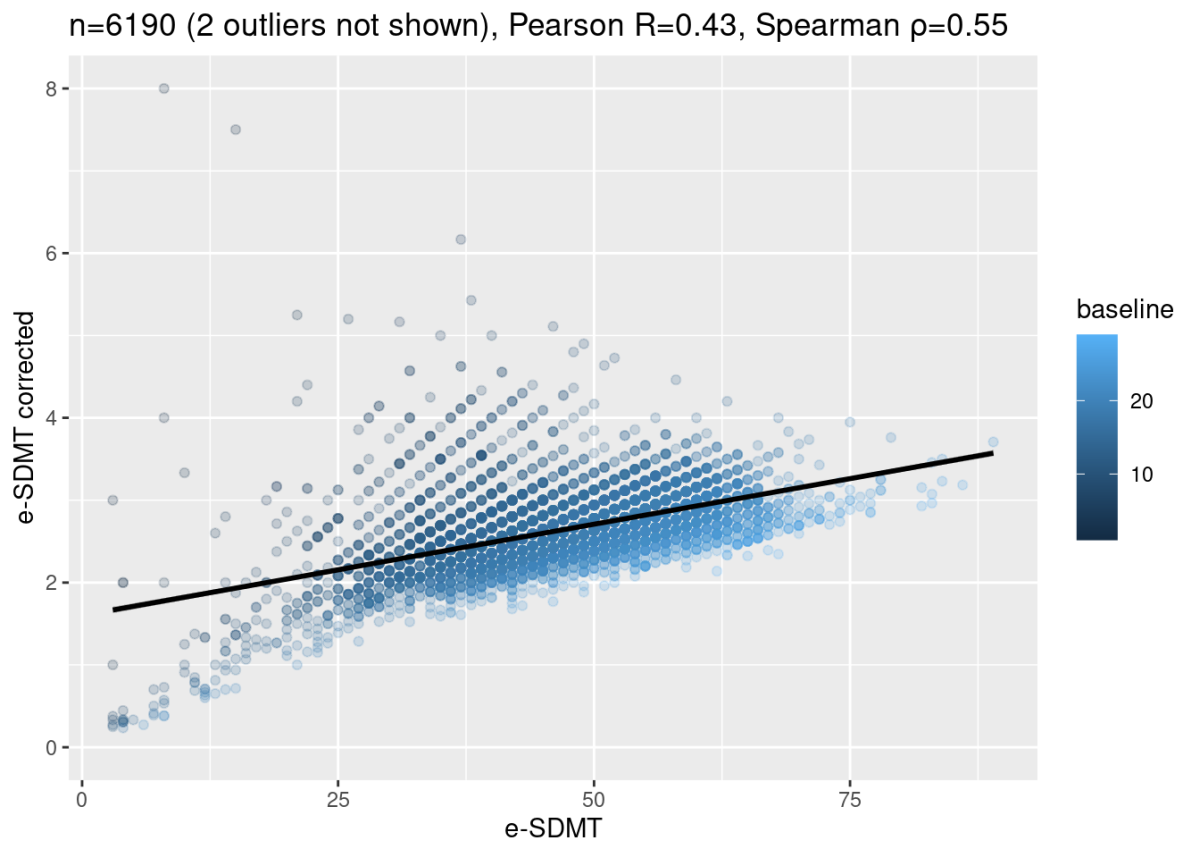

Supplement: Multimedia Appendix 3 [file jmir_v23i11e30394_app3.png]

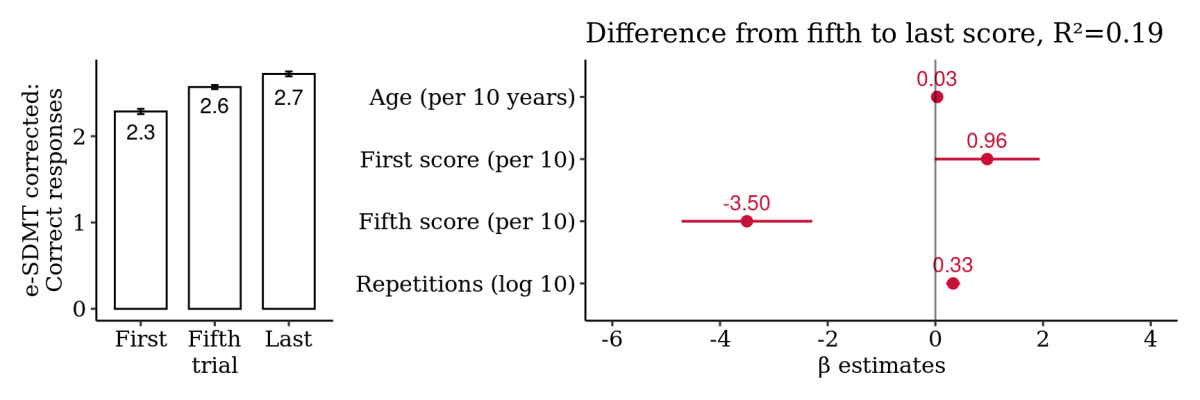

Supplement: Multimedia Appendix 4 [file jmir_v23i11e30394_app4.png]

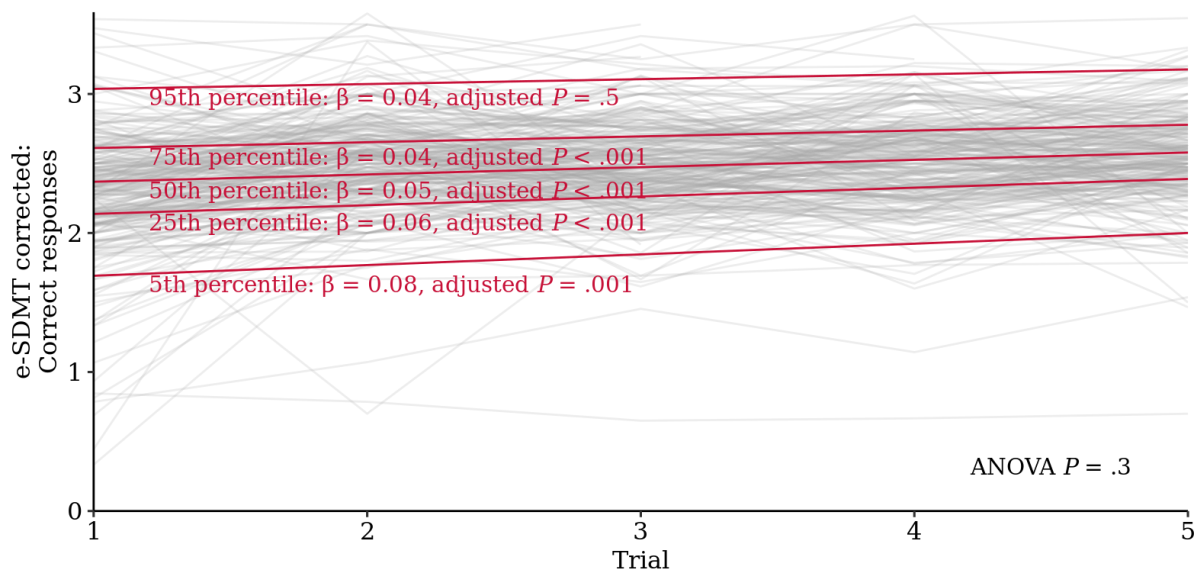

Supplement: Multimedia Appendix 5 [file jmir_v23i11e30394_app5.png]

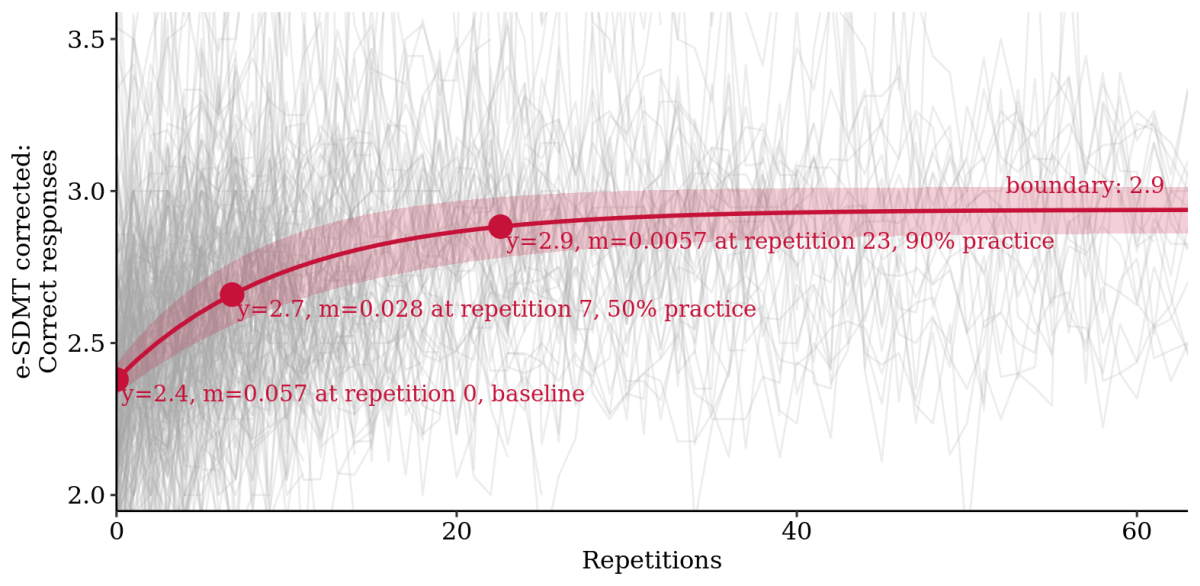

Supplement: Multimedia Appendix 6 [file jmir_v23i11e30394_app6.png]

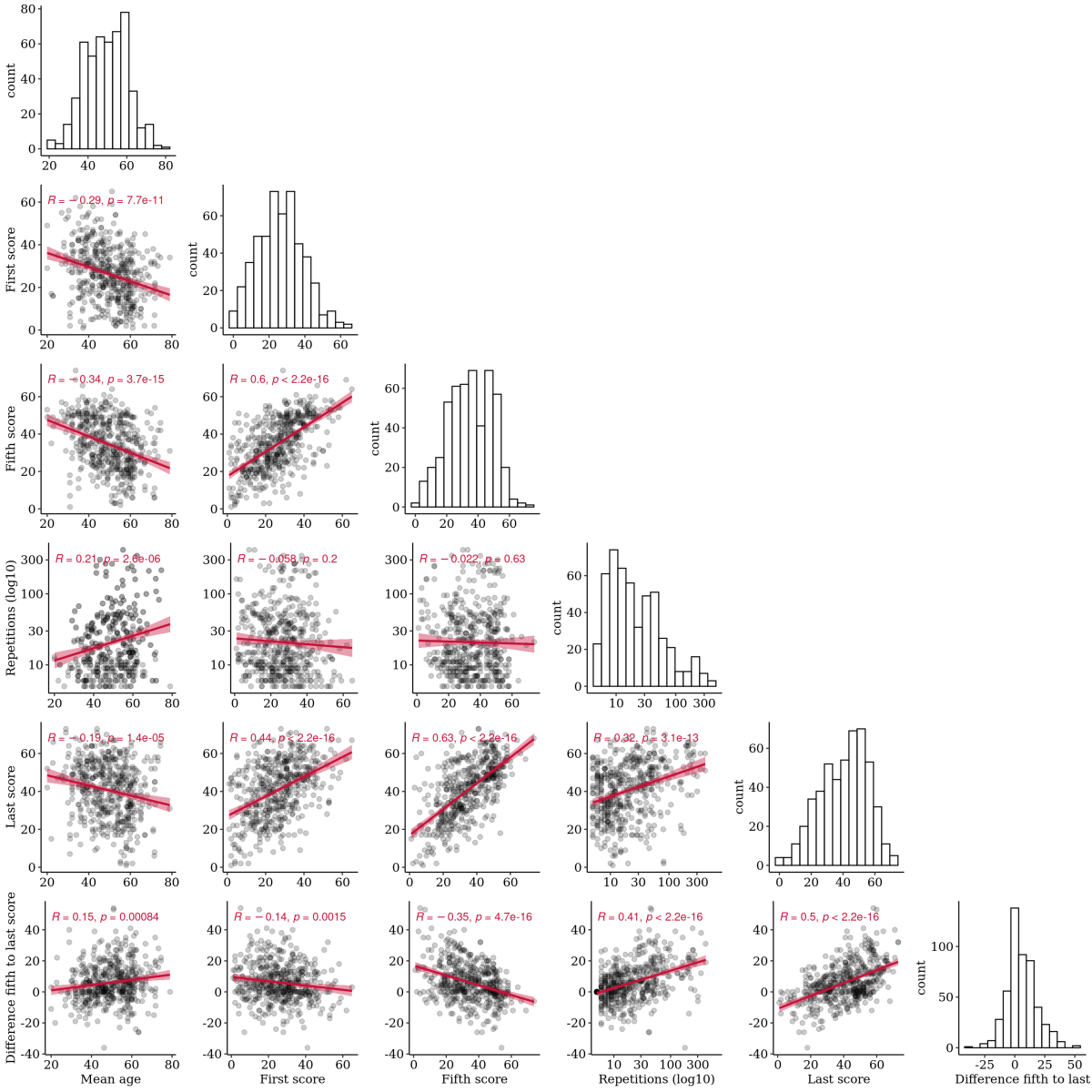

Supplement: Multimedia Appendix 7 [file jmir_v23i11e30394_app7.png]

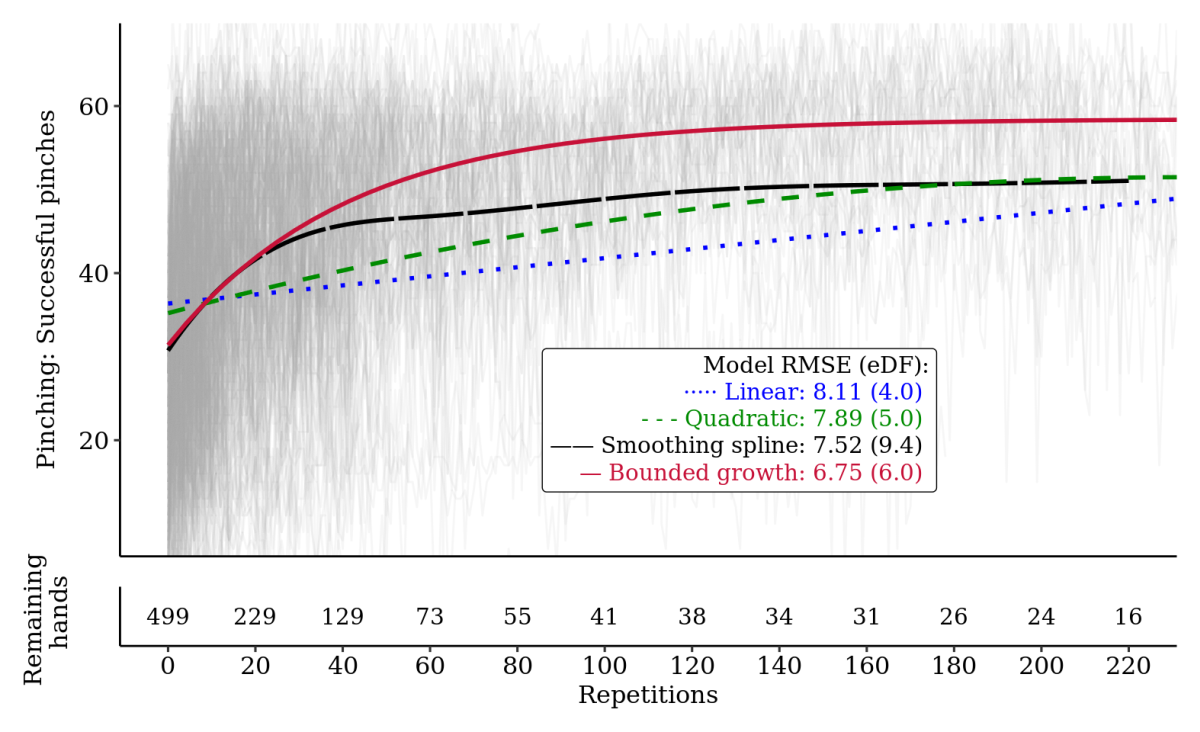

Supplement: Multimedia Appendix 8 [file jmir_v23i11e30394_app8.png]

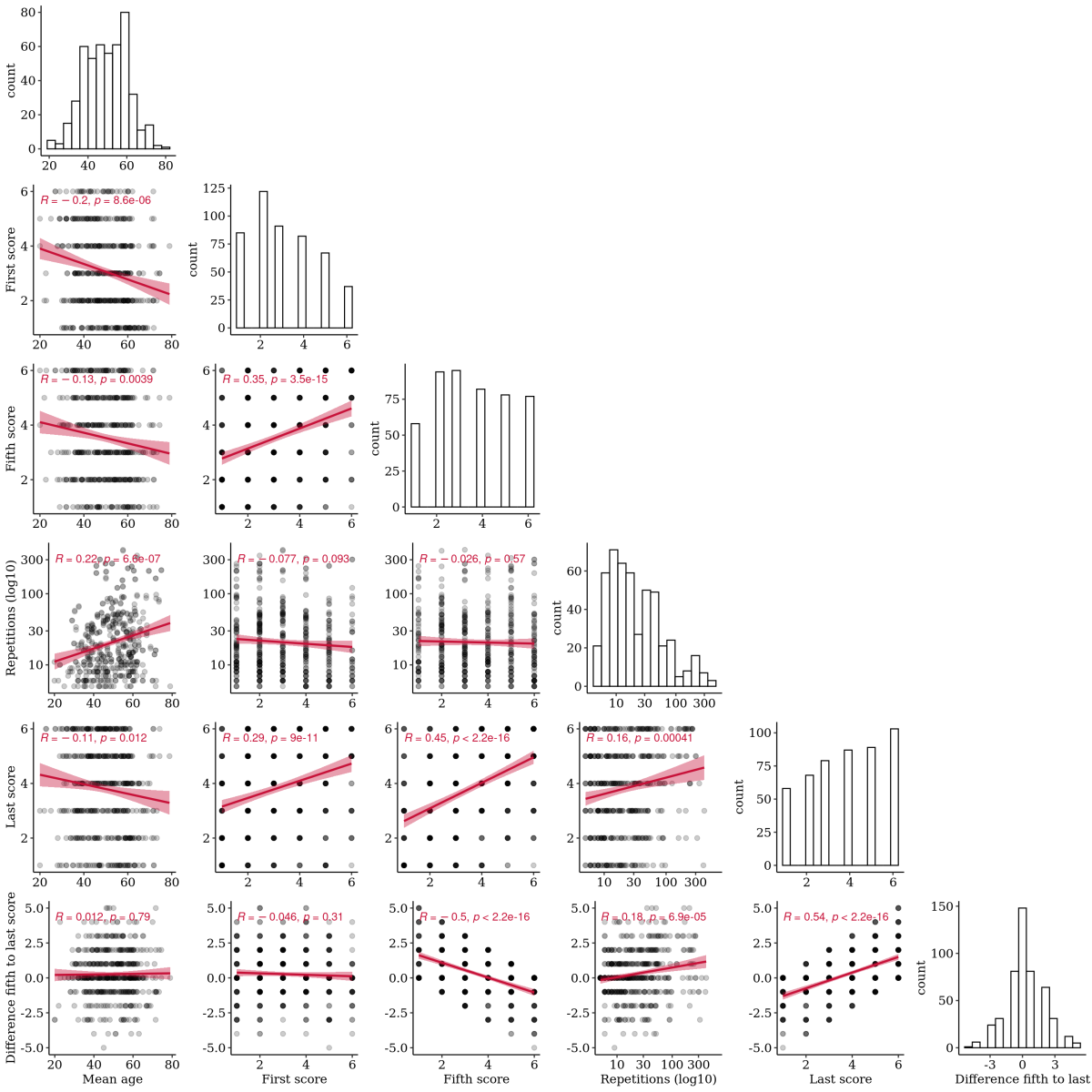

Supplement: Multimedia Appendix 9 [file jmir_v23i11e30394_app9.png]

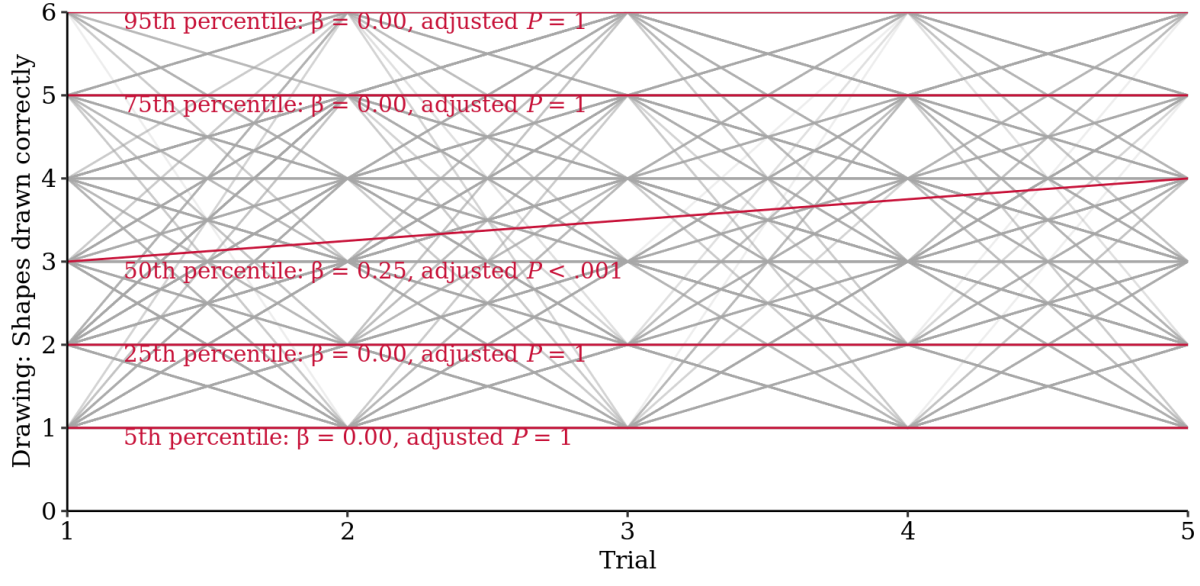

Supplement: Multimedia Appendix 10 [file jmir_v23i11e30394_app10.png]

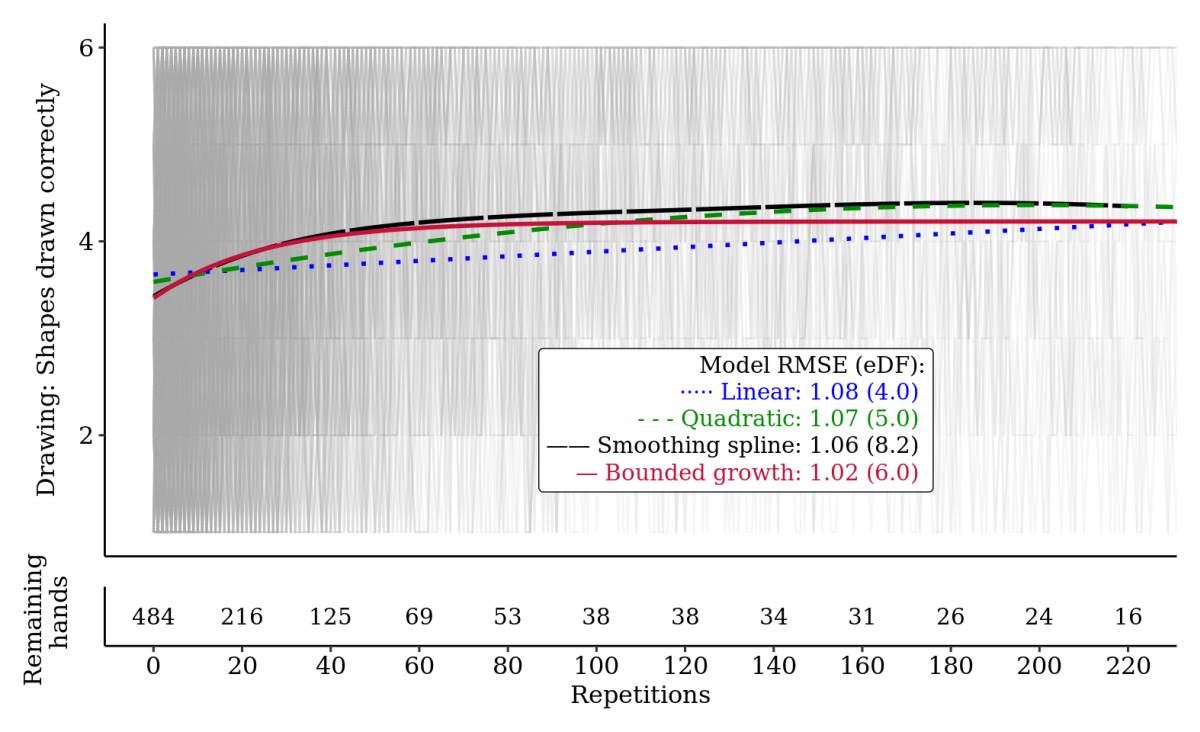

Supplement: Multimedia Appendix 11 [file jmir_v23i11e30394_app11.png]

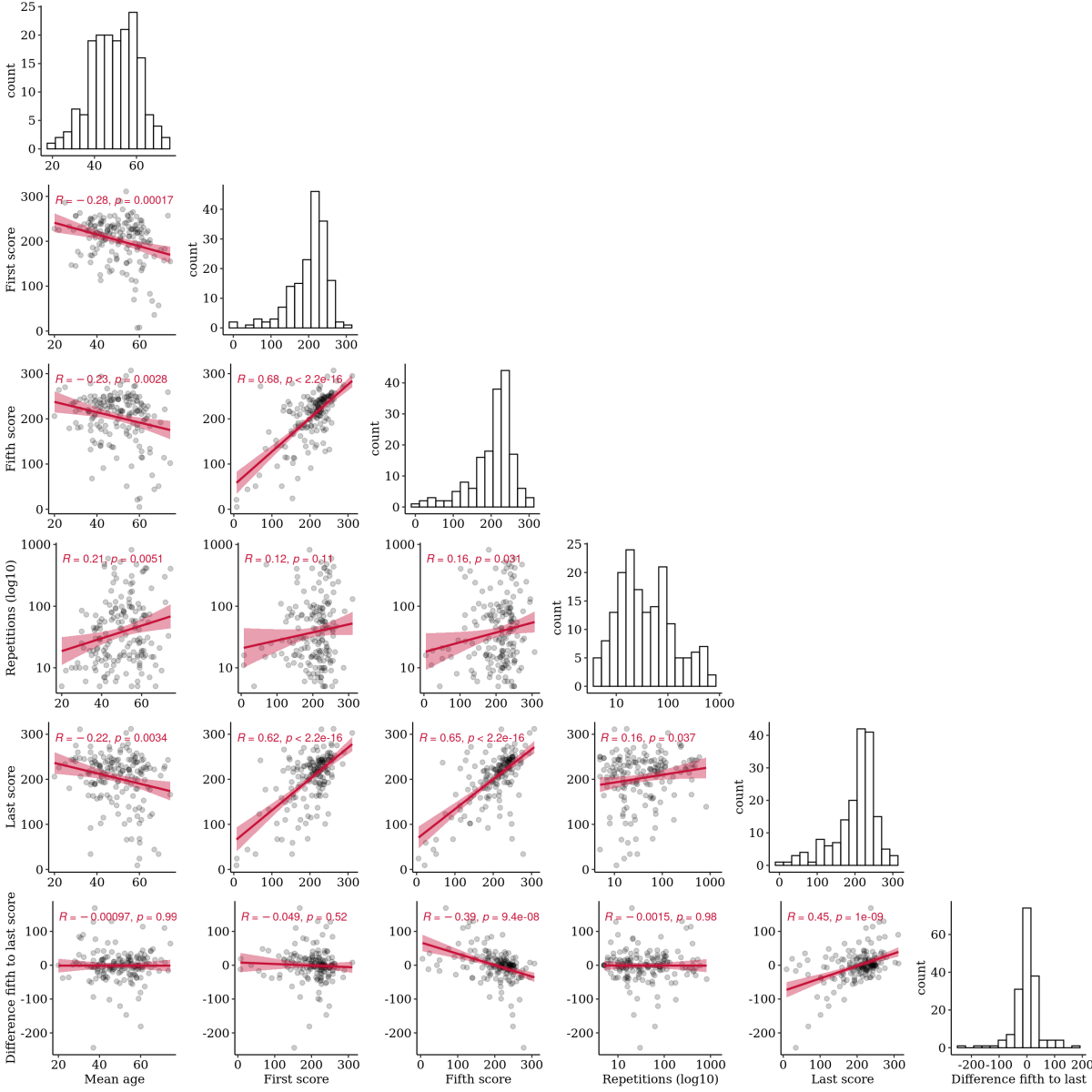

Supplement: Multimedia Appendix 12 [file jmir_v23i11e30394_app12.png]

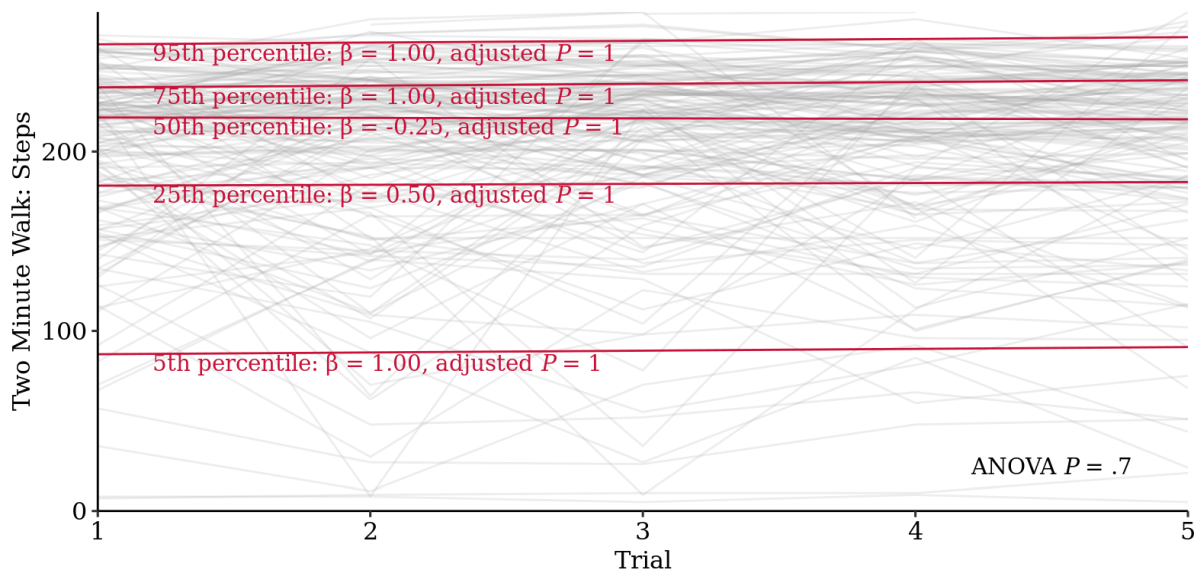

Supplement: Multimedia Appendix 13 [file jmir_v23i11e30394_app13.png]

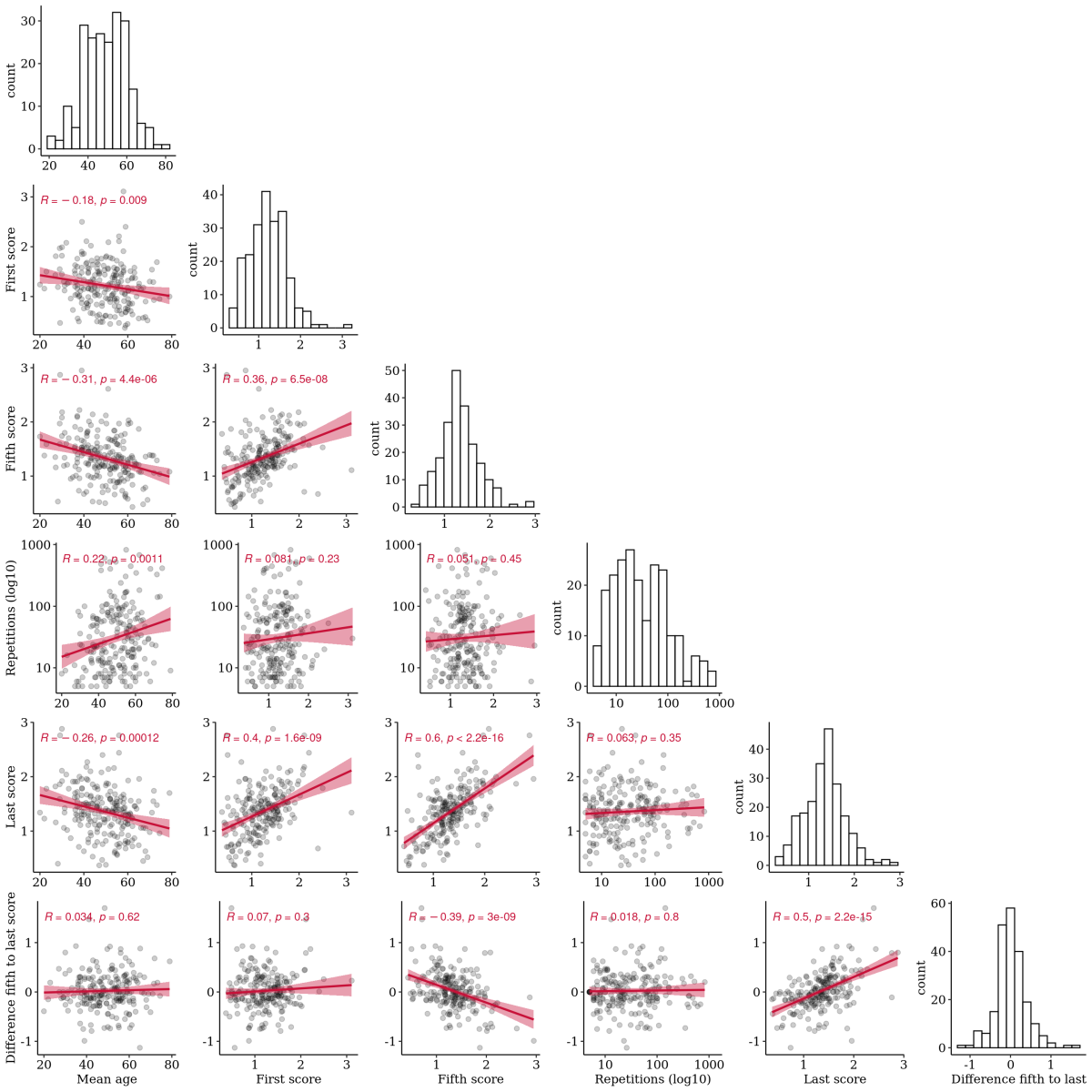

Supplement: Multimedia Appendix 14 [file jmir_v23i11e30394_app14.png]

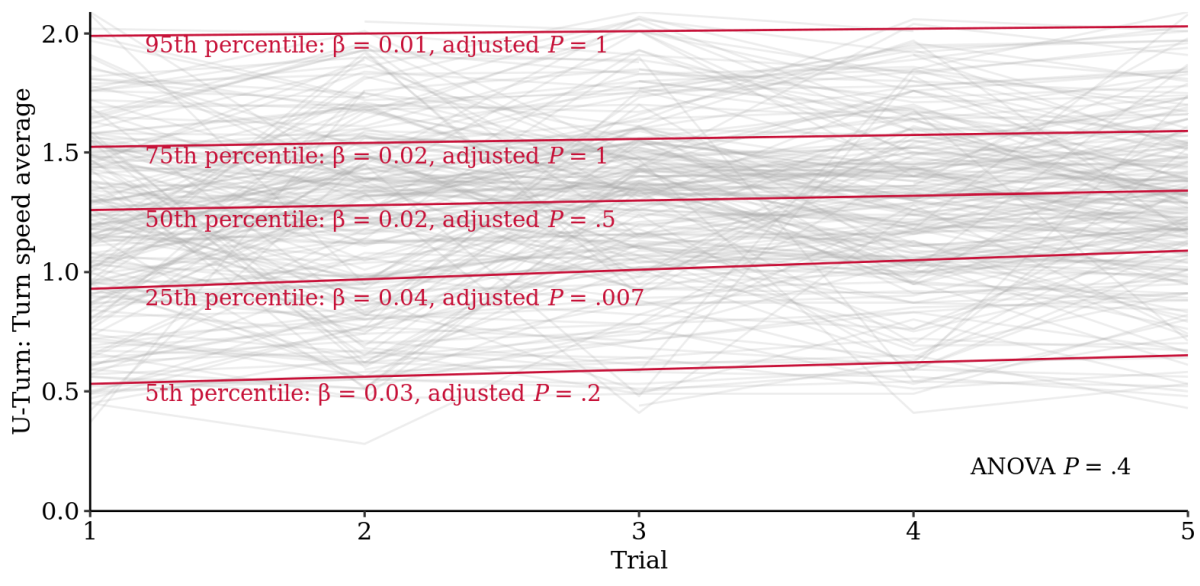

Supplement: Multimedia Appendix 15 [file jmir_v23i11e30394_app15.png]

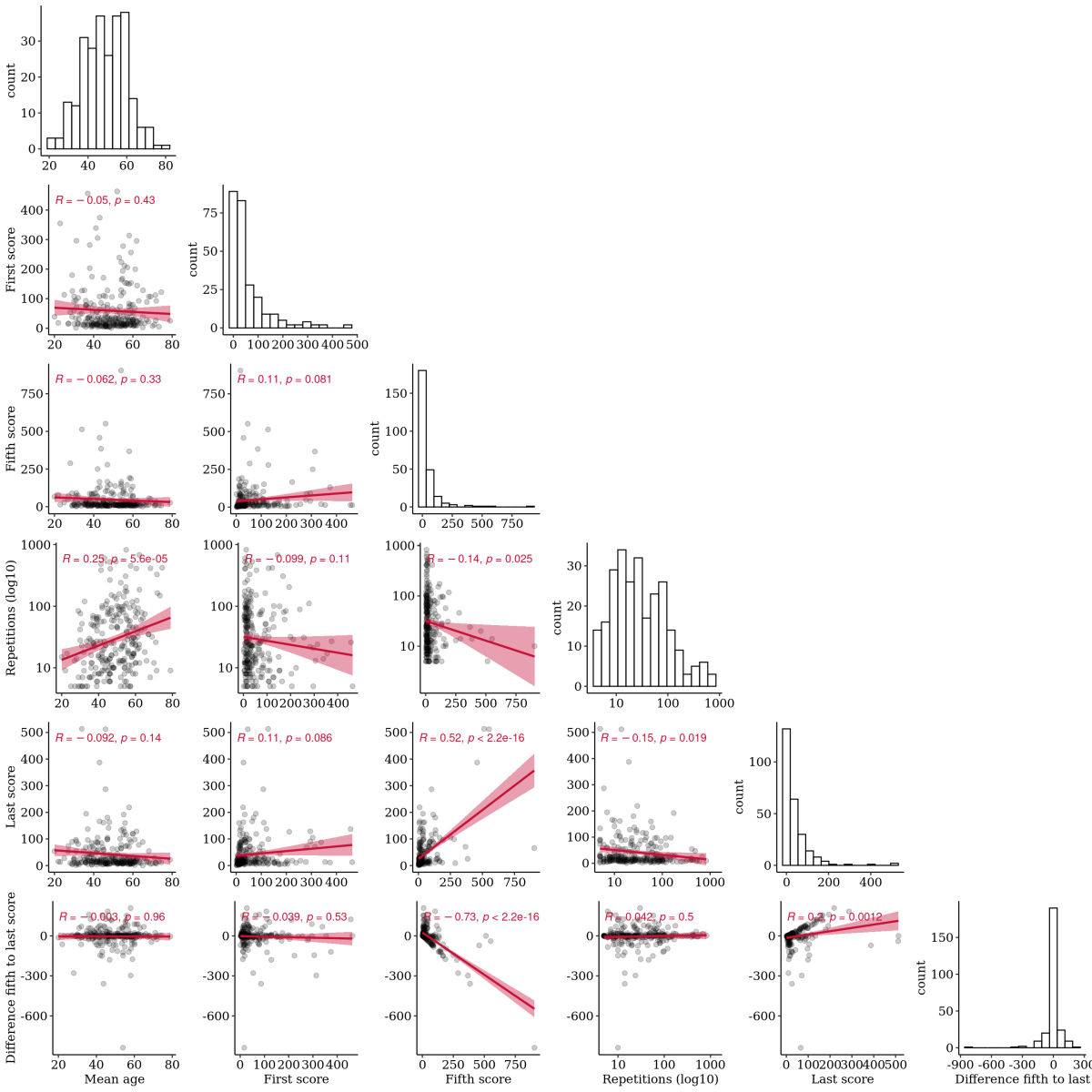

Supplement: Multimedia Appendix 16 [file jmir_v23i11e30394_app16.png]

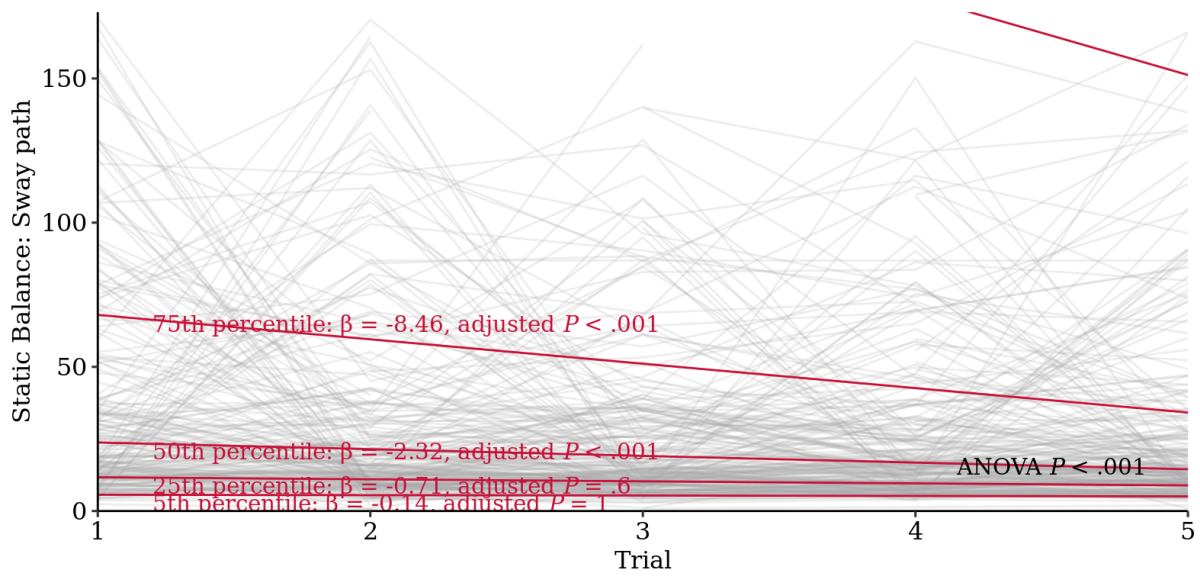

Supplement: Multimedia Appendix 17 [file jmir_v23i11e30394_app17.png]
