# Supplementary material for: Practice Effects of Mobile Tests of Cognition, Dexterity, and Mobility on Patients With Multiple Sclerosis: Data Analysis of a Smartphone-Based Observational Study
Source: J Med Internet Res. 2021 Nov 18;23(11):e30394. doi: 10.2196/30394 (PMC8663564; doi:10.2196/30394)
Supplement: Multimedia Appendix 18 [file jmir_v23i11e30394_app18.pdf]

doi:10.2196/30394

Practice Effects of Mobile Tests of Cognition, Dexterity, and Mobility on Patients With Multiple Sclerosis: Data Analysis of a Smartphone-Based Observational Study

Tim Woelfle, Silvan Pless, Andrea Wiencierz, Ludwig Kappos, Yvonne Naegelin, Johannes Lorscheider

Multimedia Appendix 18: Key results for the main analysis vs sensitivity analyses 1-3 for cognition, dexterity, and mobility.

	**Minimum of 5 weeks & 5 repetitions**		**Minimum of 10 weeks & 10 repetitions**	
	**Practice effects as a function of number of repetitions**	**Practice effects as a function of weeks since first test**	**Practice effects as a function of number of repetitions**	**Practice effects as a function of weeks since first test**
	**Main analysis**	**Sensitivity analysis 1**	**Sensitivity analysis 2**	**Sensitivity analysis 3**
**Cognition** ***e-SDMT***	n=262 patients		n=144 patients	
Mean improvement first to last score	25.4% (23.1%-27.8%)		29.1% (26.2%-32.0%)	
Mean improvement fifth to last score	5.7% (4.1%-7.4%)		9.1% (6.8%-11.4%)	
Association with difference from fifth to last-score	$\beta_{Repetitions (log10)}$ = 6.45 (4.86-8.05)	$\beta_{Weeks (log10)}$ = 3.77 (2.32-5.23)	$\beta_{Repetitions (log10)}$ = 8.15 (5.34-10.96)	$\beta_{Weeks (log10)}$ = 4.92 (2.57-7.26)
	n=4824 tests		n=4036 tests	
Boundary increase in performance	40.8%	29.6%	34.3%	27.9%
Half-practice point	repetition 11	week 12	repetition 12	week 13
**Dexterity** ***Finger Pinching***	n=499 hands		n=305 hands	
Mean improvement first to last score	54.2% (49.3%-59.1%)		68.7% (62.1%-75.3%)	
Mean improvement fifth to last score	22.7% (18.6%-26.8%)		35.0% (29.3%-40.8%)	
Association with difference from fifth to last-score	$\beta_{Repetitions (log10)}$ = 11.53 (9.44-13.62)	$\beta_{Weeks (log10)}$ = 6.87 (4.30-9.45)	$\beta_{Repetitions (log10)}$ = 12.03 (8.70-15.36)	$\beta_{Weeks (log10)}$ = 4.23 (0.07-8.38)
	n=19,650 tests		n=17,765 tests	
Model increase in performance	86.2%	50.4%	73.8%	51.9%
Half-practice point	repetition 28	week 11	repetition 29	week 12
**Dexterity** ***Draw a Shape***	n=484 hands		n=301 hands	
Mean improvement first to last score	23.9% (18.3%-29.5%)		30.3% (22.9%-37.7%)	
Mean improvement fifth to last score	8.8% (3.8%-13.8%)		13.4% (6.5%-20.2%)	
Association with difference from fifth to last-score	$\beta_{Repetitions (log10)}$ = 0.74 (0.45-1.04)	$\beta_{Weeks (log10)}$ = 0.61 (0.27-0.95)	$\beta_{Repetitions (log10)}$ = 1.02 (0.57-1.47)	$\beta_{Weeks (log10)}$ = 0.59 (0.05-1.13)
	n=19,019 tests		n=17,303 tests	

doi:10.2196/30394

Model increase in performance	23.1%	14.2%	19.8%	12.9%
Half-practice point	repetition 17	week 7	repetition 19	week 7
**Mobility** ***Two Minute Walk***	n=171 patients		n=125 patients	
Mean improvement first to last score	-0.1% (-3.5% to 3.4%)		0.0% (-4.1% to 4.1%)	
Mean improvement fifth to last score	-0.5% (-4.0% to 3.0%)		-2.1% (-6.4% to 2.1%)	
Association with difference from fifth to last-score	$\beta_{\text{Repetitions (log10)}}$ = 6.56 (-5.79 to 18.92)	$\beta_{\text{Weeks (log10)}}$ = 7.32 (-9.45 to 24.10)	$\beta_{\text{Repetitions (log10)}}$ = 11.04 (-5.20 to 27.27)	$\beta_{\text{Weeks (log10)}}$ = 12.93 (-11.14 to 37.01)
**Mobility** ***U-Turn***	n=217 patients		n= 141 patients	
Mean improvement first to last score	11.0% (5.7%-16.2%)		9.3% (3.4%-15.2%)	
Mean improvement fifth to last score	1.9% (-2.3% to 6.1%)		1.9% (-3.6% to 7.4%)	
Association with difference from fifth to last-score	$\beta_{\text{Repetitions (log10)}}$ = 0.03 (-0.06 to 0.13)	$\beta_{\text{Weeks (log10)}}$ = 0.04 (-0.08 to 0.16)	$\beta_{\text{Repetitions (log10)}}$ = 0.03 (-0.09 to 0.16)	$\beta_{\text{Weeks (log10)}}$ = 0.13 (-0.05 to 0.30)
**Mobility** ***Static Balance***	n= 257 patients		n=165 patients	
Mean improvement first to last score	-28.6% (-48.6% to -8.5%)		-32.3% (-57.5% to -7.2%)	
Mean improvement fifth to last score	-7.5% (-24.1% to 9.2%)		-16.6% (-42.1% to 8.9%)	
Association with difference from fifth to last-score	$\beta_{\text{Repetitions (log10)}}$ = -7.58 (-21.28 to 6.13)	$\beta_{\text{Weeks (log10)}}$ = -10.86 (-28.12 to 6.41)	$\beta_{\text{Repetitions (log10)}}$ = -8.54 (-25.60 to 8.53)	$\beta_{\text{Weeks (log10)}}$ = -0.98 (-24.18 to 22.22)
